# Heavy metals toxicity in plants: understanding mechanisms and developing coping strategies for remediation: a review

**DOI:** 10.1186/s40643-025-00930-4

**Published:** 2025-09-04

**Authors:** Heba I. Mohamed, Izhar Ullah, Muhammad Danish Toor, Nouraiz Ahmed Tanveer, Muhammad Mughees Ud Din, Abdul Basit, Yaqoob Sultan, Murad Muhammad, Muneeb Ur Rehman

**Affiliations:** 1 Department of Biological and Geological Sciences, Faculty of Education, Ain Shams University, Cairo, 11341 Egypt; 2 Department of Horticulture, Faculty of Agriculture, Ondokuz Mayıs University, Samsun, Turkey; 3 Department of Soil Science and Plant Nutrition, Ondokuz Mayis University, Samsun, Turkey; 4 Department of Agricultural Biotechnology, Faculty of Agriculture, Ondokuz Mayıs University, Samsun, Turkey; 5 Institute of Soil and Environmental Sciences, University of Agriculture, Faisalabad, Pakistan; 6 Department of Horticulture Science, Kyungpook National University, 41566 Daegu, South Korea; 7 Department of Grass Breeding, Institute of Agriculture, Lithuanian Research Centre for Agriculture and Forestry, 58344 Kedainiu˛ r., Lithuania; 8 State Key Laboratory of Desert and Oasis Ecology, Key Laboratory of Ecological Safety and Sustainable Development in Arid Lands, Xinjiang Institute of Ecology and Geography, Chinese Academy of Sciences, Urumqi, 830011 People’s Republic of China; 9 University of Chinese Academy of Sciences, Beijing, 100049 People’s Republic of China; 10 Xinjiang Key Laboratory of Biodiversity Conservation and Application in Arid Lands, Xinjiang 10 Institute of Ecology and Geography, Chinese Academy of Sciences, Beijing, China; 11 Department of Agrochemistry and Soil Science, Faculty of Agronomy, Agricultural University, Plovdiv, Bulgaria

**Keywords:** Heavy metal, Remediation techniques, Soil health and ecosystem, Sustainable agriculture

## Abstract

Heavy metal (HM) contamination is an increasing environmental and agricultural concern due to the persistence, toxicity, and bioaccumulative nature of metals such as cadmium (Cd), lead (Pb), mercury (Hg), and arsenic (As). These pollutants are primarily introduced through industrial effluents, mining, and agrochemicals, negatively impacting soil health, crop productivity, and food safety, ultimately posing serious risks to both ecosystems and human health. Conventional remediation methods can be costly, labor-intensive, and environmentally disruptive. Heavy metals like Cd, Pb, Hg, and As disrupt cellular homeostasis, inhibit photosynthesis, generate oxidative stress, and interfere with nutrient uptake, leading to significant yield losses in plants. In response to these stresses, plants utilize complex molecular mechanisms for tolerance, including the activation of antioxidant enzymes, upregulation of metal transporters, production of metal-chelating molecules, and modulation of stress-responsive genes and transcription factors. In contrast, bioremediation offers a sustainable and eco-friendly alternative by leveraging the detoxification capabilities of plants, microbes, and their symbiotic interactions. Techniques such as phytoremediation, microbial-assisted remediation, and integrated strategies involving biochar and organic amendments have demonstrated promising results in restoring heavy metal-contaminated soils. Recent advancements in molecular biology and synthetic biology have further improved the efficiency of bioremediation through the genetic engineering of hyperaccumulator plant species and metal-resistant microbes. This review examines the toxic effects of heavy metals on plants and highlights innovative, nature-based remediation strategies, emphasizing their potential for scalable and sustainable environmental cleanup.

**Graphical Abstract:**

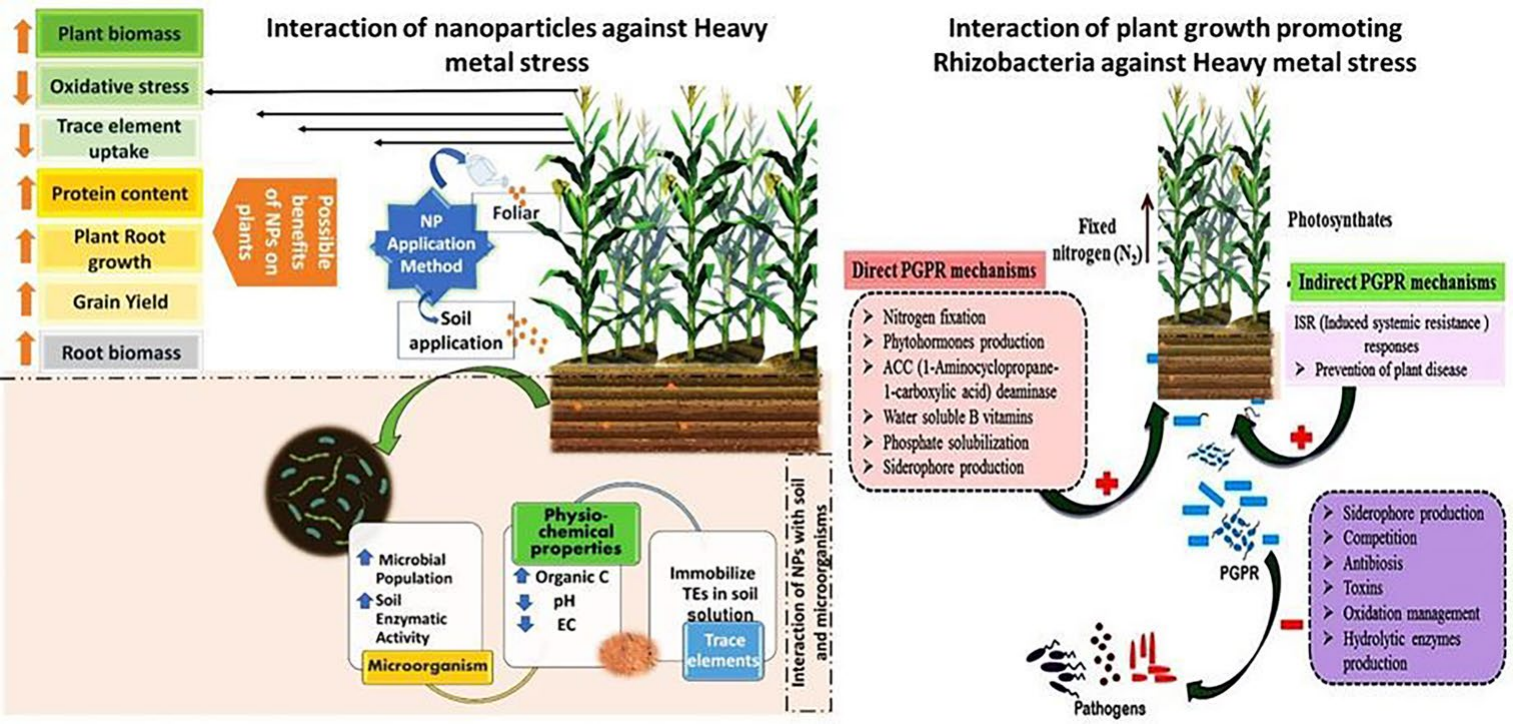

## Introduction

Environmental pollution is a major obstacle to sustainable agriculture. Soil and water can become contaminated by heavy metals, pesticides, and excess fertilizers, harming soil health, reducing biodiversity essential for pest control and pollination, and posing food safety risks. Pollution also exacerbates climate change, with extreme weather events devastating crop yields and water resources. Much of this degradation originates from point source pollution linked to mining, foundries, smelters, and other metal industries (Okereafor et al. 2020). Heavy metals, characterized by a density greater than water (Zamora-Ledezma et al. 2021), significantly impact sustainable agriculture. They enter the environment through corrosion, atmospheric deposition, erosion, groundwater leaching, sediment resuspension, and water evaporation (MathuMitha et al. 2021). Manufacturing in the plastics, textiles, microelectronics, wood preservation, and paper industries, along with operations in nuclear power plants, electrical lines, coal and petroleum combustion, and metal refining, are key industrial sources of heavy metals (Dey et al. 2021; Chaitanya et al. 2024).

Heavy metals (HMs) like Cd, Pb, As, Hg, zinc (Zn), and chromium (Cr) are highly toxic environmental pollutants that significantly impact plant growth and development (Ali et al. 2022a). These metals accumulate in soils from human activities such as industrial discharge and mining, disrupting physiological and biochemical processes in plants. One immediate effect of heavy metals is the inhibition of seed germination and root elongation due to osmotic stress and cell membrane damage. For example, cadmium and lead hinder water uptake and reduce essential hydrolytic enzyme activity (Nogueira et al. 2021). They also impair photosynthesis by affecting chlorophyll biosynthesis and the structure of chloroplasts, leading to stunted growth and reduced biomass (Yadav et al. 2020). Heavy metals increase the production of reactive oxygen species (ROS), causing oxidative stress and damage to cellular components. Plants respond by activating antioxidant defenses, including enzymatic such as superoxide dismutase (SOD), catalase (CAT), and ascorbate peroxidase (APX) and non-enzymatic (e.g., glutathione, ascorbate) mechanisms, although their effectiveness varies by metal type and concentration (Hasanuzzaman et al. 2022). At the molecular level, heavy metals influence gene expression related to stress responses, upregulating genes for metal transporters and stress-related transcription factors (Zhou et al. 2023). They also disrupt nutrient uptake, leading to deficiencies in essential elements like iron and zinc, which weaken the plants' overall stress tolerance (Ali et al. 2022b). Moreover, long-term exposure can alter plant-microbiome interactions, reducing beneficial microbial populations and impacting nutrient cycling and disease resistance.

Traditional remediation techniques like soil excavation, washing, and chemical stabilization are often costly, disruptive, and not suitable for large-scale applications. In contrast, bioremediation—which utilizes living organisms such as microbes, plants, or their combinations to detoxify polluted environments—presents an eco-friendly and cost-effective alternative. Phytoremediation, a plant-based approach, employs mechanisms like phytoextraction, phytostabilization, and phytovolatilization to remove or immobilize metals from the soil (Ahmad et al. 2021). Similarly, microbes, particularly plant growth-promoting rhizobacteria (PGPR) and fungi, play an essential role in enhancing plant tolerance to heavy metals by producing substances such as siderophores, organic acids, and biosurfactants. They also help alter metal bioavailability (Gupta et al. 2023). The use of biochar, compost, and microbial inoculants in combination with tolerant plants has been shown to improve remediation efficiency in field conditions (Mosa et al. 2022). Recent advancements in molecular biology and synthetic biology have made it possible to genetically engineer hyperaccumulator plants and microbes with enhanced capabilities for metal uptake and tolerance. This offers new directions for innovative bioremediation strategies (Sharma et al. 2024a, b). Therefore, integrating bioremediation with modern biotechnology and sustainable agricultural practices represents a promising and scalable solution to the heavy metal pollution crisis.

Numerous reviews have examined the general mechanisms of heavy metal (HM) toxicity and the strategies for phytoremediation or bioremediation (Ali et al. 2013; Riyazuddin et al. 2022). However, these reviews often lack integration of recent molecular insights, such as the signaling pathways, gene expression modulation, and epigenetic responses involved in plant tolerance to heavy metals. Additionally, there is limited discussion on how plants integrate multiple defense mechanisms, including antioxidant systems, transporter regulation, hormonal cross-talk, and microbial interactions, under varying environmental conditions. Another significant gap is the insufficient comparative analysis of emerging eco-friendly remediation strategies, such as the use of plant growth-promoting rhizobacteria (PGPR), and nanomaterials. This is particularly relevant in terms of their synergistic and sustainable application in real-world conditions (Kumar and Maiti 2015; Malik et al. 2022). This review aims to address these gaps by providing a comprehensive, multidisciplinary synthesis of plant responses to heavy metal stress. It will emphasize recent molecular and biotechnological advances, and evaluate innovative remediation strategies with a focus on integration and practical applicability.

## Sources and eco-toxicity of heavy metal

**Fig. 1:**
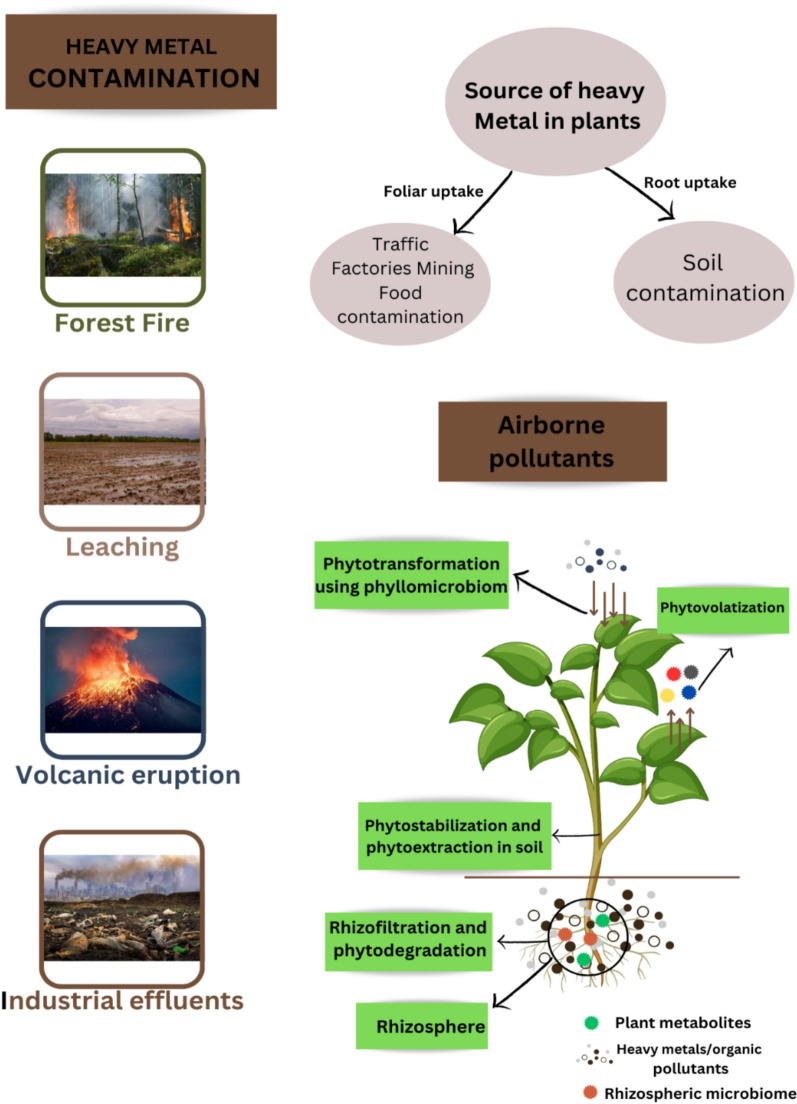
Sources of heavy metals

There are two types of sources of heavy metals in the environment: anthropogenic and natural (geogenic or lithogenic) as shown in Fig. 1. Natural activities that release metals into the environment include volcanic eruptions and the weathering of rocks that contain metals (Ali et al. 2019). Global developments in urbanization and industrialization have significantly increased the impact of human activities to environmental heavy metal contamination (Li et al. 2020). Mining, industrial operations, and agricultural practices are among the major anthropogenic causes that have been identified. When the elements are recovered from their respective ores or during the mining process, heavy metals are released (Pujari and Kapoor 2021). Both wet and dry atmospheric deposition processes make it easier for materials to get onto land (Sobhanardakani 2019). Heavy metals are released into the environment through wastewater discharge, including household and industrial effluents. Wastewater discharge, including both home and industrial effluents, contributes to elevated levels of heavy metals in the environment. The use of chemical fertilizers and the burning of fossil fuels are two examples of human actions that introduce heavy metals into the environment. One type of fertilizer that is distinguished by a particular heavy metal content is phosphate fertilizer. Secondary sources such as fertilizers, herbicides, and insecticides raise the concentration of heavy metals in agricultural soils over time (Alengebawy et al. 2021). Volcanic activity, metal corrosion, soil and water evaporation, sediment resuspension, soil erosion, and geological weathering are among the variables that contribute to heavy metal contamination (Nachana’a Timothy 2019; MathuMitha et al. 2021).

Phosphate fertilizers produced from acidulated phosphate rock (PR) represent a significant source of heavy metals in agricultural soils. Sulphuric acid is utilized in the production of single superphosphate (SSP) and phosphoric acid in the production of triple superphosphate (TSP) (Ahmad et al. 2019). The fertilizers produced from phosphate rock retain all the heavy metals present in the rock (Viana et al. 2024). Commercial inorganic fertilizers, especially those containing phosphate, are significant contributors to the global dispersion of heavy metals (Qu et al. 2024). The application of heavy metals to agricultural soil has the potential to leach into groundwater, resulting in contamination (Srivastav et al. 2020).

## Mechanism of heavy metals’ uptake, translocation, tolerance, and molecular pathways

### Heavy metals uptake

**Fig. 2:**
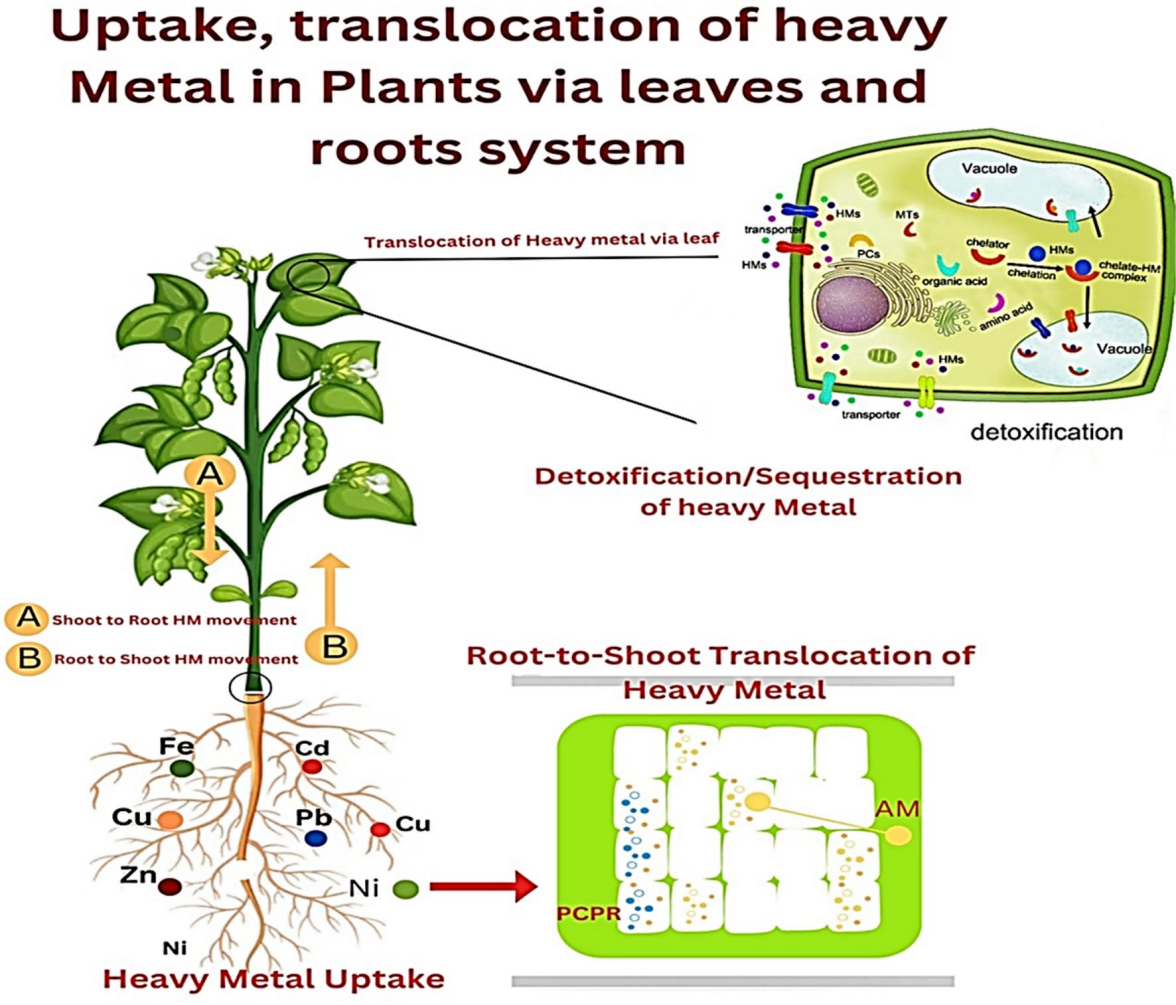
Mechanism of heavy metals’ uptake, translocation, and tolerance in plants. Arbuscular mycorrhizal (AM)

Hyperaccumulators possess a distinctive capability to uptake significantly elevated levels of heavy metals from soil, even amidst fluctuating concentrations of these metals (Shrivastava et al. 2019). Hyperaccumulators are a unique group of plant species capable of thriving in metal-contaminated environments by absorbing and storing exceptionally high concentrations of heavy metals in their aerial parts, often without exhibiting toxicity symptoms. This exceptional ability makes them valuable in phytoremediation, particularly for phytoextraction strategies (Shrivastava et al. 2019). Hyperaccumulators have the ability to accumulate heavy metals; however, their uptake is influenced by various factors, including pH, water content, and organic substances, among others. Additionally, an operational transport system within the plant is essential for an effective heavy metal uptake mechanism, as illustrated in Fig. 2. Numerous investigations indicate that pH influences the absorption of heavy metals in hyperaccumulators through two primary mechanisms: (i) by promoting the dissolution of metals via proton secretion from the roots, which acidifies the rhizosphere, and (ii) by affecting the growth of plant species that accumulate metals (Ma et al. 2016). It has been shown that, alongside pH, the organic substances released from the roots play a crucial role in the growth of hyperaccumulating plants. According to Islam et al. (2024), the organic acids generated by the roots played a role in affecting Cd solubility through the creation of Cd complexes. Consequently, the pH levels and organic compounds released from the rhizosphere facilitate and improve the absorption of heavy metals (Seshadri et al. 2015). Moreover, a significant absorption of heavy metals is frequently associated with greater root proliferation, which is believed to promote improved metal uptake (Chen et al. 2024a, b).

### Heavy Metals Translocation from Root to Shoot

In contrast to non-hyperaccumulator plants, hyperaccumulators do not hold onto the heavy metals taken up by their roots; instead, they transport these metals through the xylem to the shoots. The process involves various classes of proteins, each playing a crucial role in the transport of heavy metals within the plant. The movement of heavy metals between the intracellular environment and cytosol is facilitated by CPx-type and P1B-type H + ATPases, as well as proteins from the Nramp, cation diffusion facilitator (CDF), zinc–iron permease (ZIP) families, and multidrug and toxin efflux (MATE) proteins (Bozzi and Gaudet 2021). The transport of ATP is facilitated by CPx-type ATPases, which primarily function to move toxic metals such as Cu, Zn, Cd, and Pb across cell membranes (Adhikary et al. 2024). P1B type ATPases play a role in the transport of heavy metals, as well as in the regulation of metal homeostasis and mechanisms of metal tolerance (Østerberg and Palmgren 2018). The increased expression of heavy metal ATPases (HMAs) in the roots and shoots of hyperaccumulators indicates that these proteins are more prevalent in hyperaccumulators than in non-hyperaccumulators (Dar et al. 2018). Natural resistance associated macrophage proteins represent a significant category of proteins that play a crucial role in metal transport. The genes in question are known as Nramp genes. Research has revealed the presence of three homologs of the Nramp protein in rice: OsNramp1, OsNramp2, and OsNramp3. The expression of these proteins in rice varies across different tissues and facilitates the transport of distinct, yet closely related, and ions (Mani and Sankaranarayanan 2022; Yu et al. 2024).

### Detoxification/sequestration of heavy metal

Shortly after the transportation of heavy metals to the shoots, hyperaccumulators effectively sequester and detoxify these metals, enabling them to thrive in contaminated environments without experiencing toxic repercussions. Detoxification and sequestration processes primarily occur within the vacuole of plant cells (Ansari et al. 2024). This process involves the action of various transporter families, including ATP-binding cassette (ABC), cation diffusion facilitator (CDF), zinc–iron permease (ZIP), heavy metal transport ATPase (CPx- and P1B-ATPase), and natural resistant associated macrophage protein (NRAMP) transporters. The primary function of ABC transporters in the transport of heavy metals was demonstrated, highlighting the involvement of two significant subfamilies: MRP (multidrug resistance associated protein) and PDR (pleiotropic drug resistance) in the active transport of heavy metals. The phytochelatin–cadmium (PC–Cd) transporting vacuolar ABC transporter, HMT1, represents the inaugural characterization of a vacuolar ABC transporter in *Schizosaccharomyces pombe*, located within the tonoplast (Singh et al. 2016). Our findings indicate that HMT1 possesses a functional homolog in both *Caenorhabditis elegans* and Drosophila (Kim et al. 2018); however, thorough investigations of homologs in plant species remain lacking. Two transporters in *Arabidopsis thaliana*, AtMRP1 and AtMRP2, were identified as participants in metal (Cd(II) and Pb(II)) resistance via vacuolar PC–Cd transport (Noctor et al. 2024).

The metal tolerance protein (MTP) family comprises transport proteins responsible for the translocation of metal cations, including Zn^2^⁺, Cd^2^⁺, Co^2^⁺, Ni^2^⁺, and Mn^2^⁺, from the cytosol to the vacuole (Das et al. 2022). The CDF family consists of four distinct groups, with subgroup I and III being particularly significant for metal tolerance (Zhao et al. 2024; Liu et al. 2024). The expression levels of MTP1 (group III), MTP8 (group I), and MTP11 (group I) are elevated in *A. halleri* and *T. caerulescens*, both of which are classified as hyperaccumulators, compared to non-hyperaccumulators (Singh et al. 2016, 2019). The constitutive high expression pattern of the AhMTP1 protein in *A. halleri* leaves was observed following the application of exogenous zinc, which aligns with the typical expression profiles of Ah out-expressed proteins (Singh et al. 2016).

### Molecular pathways, signaling cascades, and gene regulation

Plants absorb a range of heavy metals from the soil. For plants to grow normally, these heavy metals may or may not be required (Li et al. 2022a). Plants' biological processes suffer irreversible damage when heavy metals continuously stress their growth and development, which lowers plant productivity and output (Li et al. 2022a). As a result, plants undergo a number of physiological and biochemical alterations in response to an adaptation to these environmental stresses. Numerous intricate signal channels are involved in this process (Kumar et al. 2020). In essence, stressors can use exterior signals (calcium or miRNA) to cause intracellular signal perception (Ding et al. 2020). Accordingly, signals detected by receptors on the cell membrane surface are gradually increased by phosphorylation and dephosphorylation before being sent to cells (Mondal et al. 2022). Signals in cells have the ability to control the expression of particular genes and activate particular effector proteins, such as kinases, enzymes, or transcriptional factors, in the nucleus or cytoplasm (Hao et al. 2021).

Different intermediate molecules' heavy metal stress signals trigger distinct transcriptional factors, which in turn cause the production of various antioxidant enzymes (Kaur et al. 2021). Among these, mitogen-activated protein kinases (MAPKs) catalyze protein phosphorylation, a crucial signal transmission mechanism in plants. Among the most important and well-preserved signaling molecules in eukaryotes are MAPKs. They coordinate cellular responses for the organism's regular growth and development downstream of the sensor/receptor (Li et al. 2022a, b). MAPKs facilitate the transmission of signals produced by ligand–receptor interactions with downstream substrates and are easily recognized by their distinctive TXY activation motif and similarity in sequence (Komis et al. 2018).

**Fig. 3:**
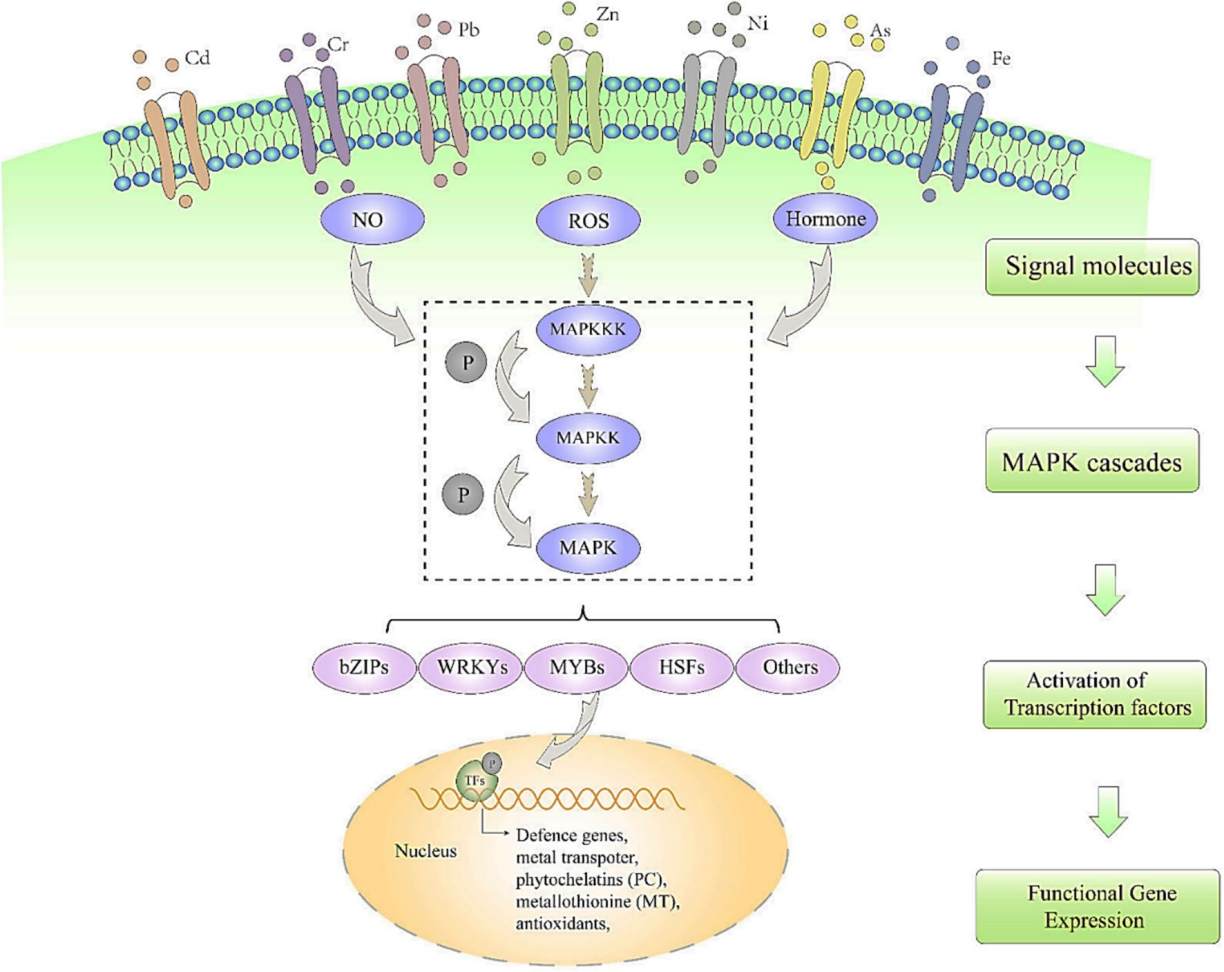
Plant responses to heavy metal stressors include transcriptional factors and the MAPK cascade (Li et al. 2022a). Numerous signaling pathways, including NO, ROS, and phytohormones, are triggered by exposure to heavy metals. The MAPK cascade is triggered by the interaction of these signals. The MAPK cascade then phosphorylates and activates related transcriptional factors, such as bZIP, WRKY, MYB, HSF, and others. This further triggers the production of genes related to defense, metal transport, PCs, MTs, antioxidants, and other processes. Lastly, plants are more able to withstand or accumulate heavy metals

There are several MAPK pathways in cells that are interconnected yet operate independently, playing vital roles in cell signal transmission. In plants, the MAPK cascade interacts with other signaling pathways, regulating growth, development, and responses to biotic and abiotic stresses (Sharma et al. 2021). However, research on MAPK activation by transcription factors under heavy metal stress is limited. Plant roots primarily absorb heavy metals from soil, and upon detecting heavy metal stress, they activate a signaling system to regulate gene transcription (Xue et al. 2020). The MAPK pathway consists of a phosphorylation series: MAPKKKs activate MAPKKs, which in turn activate MAPKs, responsible for phosphorylating transcription factors that induce metal response genes (Li et al. 2022a, b). Each MAPK cascade level is encoded by a small gene family, allowing for redundancy, and plant MAPKs are typically located in the cytoplasm and/or nucleus, with some being transported between these compartments (Zhang and Zhang 2022). Understanding how signal transduction and regulatory pathways develop in plants under heavy metal stress is crucial. Therefore, we concentrated on the molecular processes by which heavy metals enter plant cells to trigger the MAPK cascade signal pathway and generate reactive oxygen species, nitric oxide, and plant hormones (Li et al. 2022a, b). Furthermore, Fig. 3 summarizes the downstream transcriptional factors that react to the genes in the MAPK cascade.

As a cell signaling enzyme, MAPK plays a crucial role in regulating various biological processes in eukaryotes. MAPK pathways are highly developed and complex, often activated in response to both biological and abiotic stressors. In plants, heavy metal stress triggers multiple signaling pathways, including the MAPK cascade. For instance, in the roots of *Broussonetia papyrifera* subjected to Cd stress, MAPK transcript levels were downregulated at 3 h but upregulated after 6 h (Xu et al. 2019). Additionally, overexpression of the OsMPK3 and OsMPK6 genes led to increased transcription of stress response genes that encode superoxide dismutase, ascorbate peroxidase, glutamine synthase, and aldehyde oxidase in the presence of arsenic and drought stress (Pandey et al. 2020). Furthermore, the SlMAPK3 gene in tomatoes was significantly induced following treatment with Cd^2^⁺. Overexpression of SlMAPK3 in transgenic plants resulted in increased leaf chlorophyll content, enhanced root biomass accumulation, and improved root activity, indicating that SlMAPK3 contributes to enhanced tolerance to Cd stress (Muhammad et al. 2019).

Signal molecules like nitic oxide (NO), active ROS, and plant hormones can interact with the MAPK cascade. In Cd-stressed rice seedlings, the interplay among ABA, auxin, MAPK signaling, and the cell cycle has also been documented (Zhao et al. 2014). Excess Cd or Cu exposure in *Arabidopsis thaliana* triggered MAPK cascades, hydrogen peroxide (H_2_O_2_) overproduction, and NADPH oxidases (Opdenakker et al. 2012). Furthermore, the roots of soybean seedlings treated with 25 mg·L^−1^ Cd exhibited elevated MAKPK2 transcription level and NO generation (Chmielowska-Bak et al. 2013).

ROS in plants is well known to be induced by heavy metals. As signaling molecules, ROS led to the activation of MAPK kinases. Two important MAPK cascades, MEKK1-MKK4/5-MPK3/6 and MEKK1-MKK2-MPK4/6, operate downstream of ROS and are involved in abiotic stress responses (Li et al. 2022a, b). Copper (Cu^2+^), as a redox-active metal, directly induces the formation of ROS at certain concentrations. In experiments with alfalfa seedlings exposed to excessive Cu^2+^, ROS accumulation was observed, which subsequently activated four different mitogen-activated protein kinases (MAPKs): SIMK, MMK2, MMK3, and SAMK (Li et al. 2022a, b). Similarly, Cd stress was found to activate ZmMPK3-1 and ZmMPK6-1 in maize roots through ROS induction (Liu et al. 2019a, b, c). In Arabidopsis seedlings treated with Cd, the activities of MPK3 and MPK6 significantly increased; however, this increase was inhibited in plants that were pretreated with the ROS scavenger glutathione (GSH). These results indicate that the accumulation of ROS induced by Cu^2+^ or Cd^2+^ in plants activates the MAPK cascade (Li et al. 2022a, b).

NO plays a crucial role in plant growth and development and regulates responses to heavy metals in plants. The interaction between NO signaling and the MAPK cascade in heavy metal-treated plants has been known for some time. When applied to the roots of Arabidopsis, NO can rapidly activate protein kinases with MAPK characteristics (Li et al. 2022a, b). In an experiment, two-week-old Arabidopsis plants were exposed to 100 µM CdCl_2_ for 24 h to investigate Cd^2+^induced NO production using the NO-sensitive fluorescent probe DAF-FM diacetate. The results showed that Cd^2+^induced MAPK and caspase-3-like activities were inhibited in the presence of the NO-specific scavenger cPTIO. This evidence suggests that NO can quickly activate protein kinases with MAPK properties in the roots of Arabidopsis (Ye et al. 2013). Conversely, caspase-3-like activity was significantly inhibited in mpk6 mutants after Cd^2+^ treatment, and these mutants exhibited reduced tolerance to both Cd^2+^ and NO compared to normal plants (Jin et al. 2013). Additionally, exposure to excessive levels of arsenic adversely affects the growth of rice seedlings, leading to increased production of ROS and NO in the roots. Following this exposure, MAPK and MPK pathways were activated in the rice leaves and roots, respectively (Rao et al. 2011).

When exposed to Cd, abscisic acid (ABA) may lessen auxin accumulation, influence auxin distribution, and partially offset the inhibitory effect of Cd on rice root growth. Furthermore, MAPK adversely regulates the important auxin signal transduction genes, such as *YUCCA, PIN, ARF*, and *IAA* (Zhao et al. 2014). Furthermore, Cd exposure increased genes linked to the ET and MAPK signal pathways in soybean seedlings. Later, promoter sequence analysis revealed that MAPKK2 included several regulatory motifs that were responsive to ethylene (ET) and other plant hormones (Chmielowska-Bąk et al. 2014).

## Mechanisms of heavy metal toxicity on plants

Heavy metals, such as Cd, Pb, As, Hg, Cr, and Ni pose significant threats to plant health by disrupting physiological, biochemical, and molecular processes (Table 1). These metals are non-biodegradable and persist in the environment, resulting in chronic toxicity for plants when present in elevated concentrations.

**Table 1:** Effect of heavy metals on plants

Heavy metals	Concentrations	Plants	Effects	References
Nickel and zinc	100 mg/kg Ni 100 mg/kg Zn	Wheat	Decreased plant growth The antioxidant enzymes superoxide dismutases (SOD), peroxidase (POD) was decreased in contaminated soil	Shahzad et al. (2024a, b)
Cadmium, chromium and lead	300 mg/kg	*Brassica chinensis* and *Brassica rapa* L	Delayed germination Decrease in germination percentage	Zunaidi et al. (2024)
Pyrene, Zinc, lead, cadmium	Pyrene (500 mg/kg), Zn (150 mg/kg), Pb (150 mg/kg), and Cd (150 mg/kg) alone and in combination	*Medicago sativa*	The growth parameters, biomass, and chlorophyll content decreased, and proline, polyphenol content and metallothionein protein content increased in the pyrene and heavy metal-polluted soils	Mathur and Panwar (2024)
Arsenic and cadmium	100 µL	Wheat	The relative growth rate and photosynthetic parameters decreased H_2_O_2_ content and lipid peroxidation increased	Direk et al. (2024)
Cadmium	20 mg Cd/kg soil	*Raphanus sativus*	Reduction in root length, shoot length, plant fresh weight, plant dry weight and chlorophyll contents	Shahzad et al. (2024a, b)
Chromium, zinc, and nickel (Ni)	2 mM	*Hordeum vulgare*	Decreased growth parameters	Belhassan et al. (2024)
Copper	Cu-contaminated soil	*Corchorus capsularis*	Reduced the seed germination, plant height, fresh biomass, photosynthetic pigment and gas exchange parameters, and increased MDA content, proline concentration and the activities of various antioxidant compounds	Ameen et al. (2023)
Copper	60, and 80 mg/kg	*Mentha arvensis*	Decreased growth parameters, photosynthetic pigments, increased antioxidant activity, reactive oxygen species production, and decreased essential oil production	Aqeel et al. (2023)
Cadmium and copper	75 µM of Cd and 20 µM of Cu	*Solanum lycopersicum*	The CAT, APX, and SOD activities increased and reduction in biomass	Saleem et al. (2023)
Chromium	100 μmol of potassium dichromate	Chinese cabbage	Reduced the content of chlorophyll a, chlorophyll b, and carotenoids, soluble protein and soluble sugar and increased MDA, H_2_O_2_, proline	Fu et al. (2023)
Chromium	50 and 100 μM	*Brassica napus*	Cr had adverse effects on plant growth, photosynthetic gas exchange attributes, leaf mesophyll ultrastructure, PSII performance and the activity of enzymatic and nonenzymatic antioxidants	Huang et al. (2024)
Aluminum	1 mM AlCl_3_	Rice	Reduced root traits, shoot attributes, and chlorophyll content	Phukunkamkaew et al. (2023)
Aluminum	200, 500, 1.000, and 2.000 μM	*Cucumis sativus*	Germination was stimulated with 500 μM of Al. Guaiacol peroxidase and ascorbate peroxidase activities were significantly stimulated catalase decreased	Kouki et al. (2021)
Lead	100, 200, 400 and 800 mg/kg	Sorghum	The germination index (GRI), radical and plumule length (cm), vigor index (VI), and tolerance index (TI) declined	Osman et al. (2023)
Cadmium	5 mg/L Cd	Lettuce	Decreased growth attributes, chlorophyll, carotenoid content, enhanced ROS, and non-enzymatic and enzymatic antioxidants	Abu-Shahba et al. (2022)
Mercury	10 and 30 mg/L	Wheat	Decreased growth parameters	Dawood et al. (2023)

### Changes in morphological parameters

Heavy metal toxicity adversely affects the overall growth performance of stressed plants, as evidenced by the confirmed reduction in growth and yield of plants cultivated in heavy metal contaminated soils (Deng et al. 2024; Li et al. 2024). The process of root growth involves complex mechanisms, including cell division and cell elongation. The presence of heavy metals leads to adverse effects on mitotic activity, resulting in inhibited root growth across various plant species (Smail et al. 2024). In the study conducted by Xu et al. (2024), it was demonstrated that the application of excess Cu^2+^ influenced root length and root morphology through alterations in auxin levels that are antagonistic to NO. Furthermore, the inhibition of root growth by heavy metals frequently increased root diameter (An et al. 2024), although this may also be influenced by the plant cytoskeleton. The results indicate that heavy metals can inhibit root growth and disrupt water balance and nutrient absorption, thereby complicating the transport of resources to aerial plant parts.

Ultimately, this negatively impacts shoot growth and biomass accumulation. This study investigates the synthesis and deposition of callose as a potential mechanism for roots to mitigate or avoid heavy metal toxicity. These mechanisms obstruct the entrapment of heavy metals, thereby facilitating root plasticity. The movement of heavy metals from soils to the aboveground parts of metallophytes, which are plants that thrive in heavy metal-contaminated soils, also includes the role of roots (Gao et al. 2024). In these plants, metals are stored in vacuoles, rendering them inactive and non-reactive.

Plant height decreased by 20% with the application of lead (Pb3000 mg/kg) and by 42% with the application of cadmium (Cd300 mg/kg) when compared to the control group. The highest dose of Pb3000 mg/kg resulted in a 15% weight loss relative to the control, while the highest dose of Cd300 mg/kg led to a significant weight loss of 63%. The accumulation rates of heavy metals in the soil, roots, and aboveground parts of the plants indicated that maize absorbed and accumulated more Cd than Pb (Elik et al. 2025). The percentage of germination, seedling length, and both fresh and dry matter significantly decreased with increasing concentrations of cadmium. Furthermore, cadmium negatively impacted plant growth, as shown by the fresh and dry weights of radish roots and leaves following treatment with 100 mg of Cd per kg of soil (Mohamed et al. 2024a, b).

### Disruption of cellular homeostasis

Heavy metals disrupt the uptake and transport of essential nutrients. For example, Cd competes with calcium (Ca^2^⁺), magnesium (Mg^2^⁺), and iron (Fe^2^⁺) for access to membrane transporters. This competition leads to nutrient imbalances and hampers metabolic functions (Singh et al. 2023). Additionally, heavy metals can displace essential cofactors in enzymes, inhibiting the enzymatic activity critical for processes such as photosynthesis, respiration, and nitrogen assimilation.

### Oxidative stress and ROS generation

One well-documented mechanism of heavy metal toxicity is the overproduction of reactive oxygen species (ROS), including hydrogen peroxide (H₂O₂), superoxide anion (O₂⁻), and hydroxyl radicals (•OH). These ROS can cause lipid peroxidation, DNA damage, and protein denaturation (Zhou et al. 2022). ROS accumulate as a result of heavy metal stress, impacting cellular redox balance. The antioxidant defense system of plants comprises enzymatic antioxidants such as superoxide dismutase (SOD), catalase (CAT), ascorbate peroxidase (APX), peroxidase (POX), glutathione reductase (GR), and glutathione peroxidase (GPX). Non-enzymatic antioxidants include ascorbate, glutathione, carotenoids, tocopherols, alkaloids, and phenolic compounds such as flavonoids and tannins. Antioxidants neutralize free radicals, mitigate oxidative damage, and safeguard cellular integrity (Meena et al. 2024). The introduction of lead (100 mg/kg soil) and cadmium (50 mg/kg soil) into cultivation soil markedly reduced several growth characteristics, such as chlorophyll, carotenoids, sugars, and proteins, while also elevating both non-enzymatic antioxidants, like phenolics and proline, and enzymatic antioxidants, such as peroxidase, superoxide dismutase, polyphenol oxidase, and catalase, in the root and shoot tissues of sugar beet plants (Badawy et al. 2024).

Applying mercury at a concentration of 9 ppm resulted in the highest activity of peroxidase (POX), showing an increase of 69.57% compared to the control group. In contrast, cadmium application at 12 ppm produced the most significant effect, increasing POX activity by 102.17% compared to the control. For superoxide dismutase (SOD), peak activity was observed at 6 ppm for both applications, with mercury application showing an increase of 84.16% and Cd application resulting in a 121.08% increase compared to the control group (Çevık et al. 2025).

### Photosynthetic impairment

Heavy metals interfere with chlorophyll biosynthesis and damage photosystem II, leading to a decrease in the photosynthetic rate. Cd and Pb are particularly recognized for hindering carbon dioxide (CO₂) fixation and degrading chlorophyll, causing chlorosis and stunted growth (Ali et al. 2021). Additionally, the inhibition of ribulose bisphosphate carboxylase/oxygenase (Rubisco) activity and disruption of chloroplast ultrastructure further increase photosynthetic limitations. Mireles-Arriaga et al. (2021) noted a decrease in growth (plumule and radicle length) in *Helianthus annuus* seedlings subjected to As. Arsenic also influenced photosynthetic pigment content, caused damage to chloroplast membranes, and disturbed nutrient balance and protein metabolism (Ahmad et al. 2020).

Cadmium treatments significantly reduced the chlorophyll and total carotenoid content in cotton plants (Farooq et al. 2016). Similarly, exposure to lead toxicity resulted in a notable decrease in chlorophyll and pigment levels in pakchoi plants (Ji et al. 2022). This reduction in photosynthesis might be attributed to the inhibition of key enzyme activities in the Calvin cycle and the photosynthetic electron transport chain, as well as the impairment of the plants’ gas exchange characteristics (Farooq et al. 2016).

### Altered hormonal balance

Exposure to heavy metals alters the balance of phytohormones, such as auxins, gibberellins, and abscisic acid. For instance, Cd stress reduces levels of indole-3-acetic acid (IAA), which inhibits root elongation, while increasing levels of ABA, leading to stomatal closure and reduced transpiration (Jorjani et al. 2024). These hormonal imbalances adversely affect both plant growth and stress responses. For example, in *Brassica juncea*, around 69 microRNAs are associated with arsenic (As) toxicity, which correlates with variations in the levels of auxins such as indole-3-acetic acid (IAA), indole-3-butyric acid (IBA), and naphthalene acetic acid (NAA), alongside changes in the expression of approximately 69 microRNAs (Ali et al. 2024). Interestingly, the external application of IAA enhanced the growth of B. juncea under arsenic stress, suggesting a role of hormonal regulation in managing arsenic-induced stress.

### Genotoxic effects

Heavy metals can induce DNA damage either through direct interaction with the DNA structure or indirectly through ROS-mediated oxidative stress. This damage can result in chromosomal aberrations, mutations, and impaired gene expression (Rahman et al. 2024). Furthermore, epigenetic changes, such as DNA methylation, can occur under heavy metal stress, potentially altering gene regulation. The study investigated the effects of chemicals on various physiological parameters, including the Mitotic Index (MI), Micro Nucleus (MN) formation, genotoxicity, and the accumulation of Co(NO_3_)_2_ in the roots. The analysis of the Mitotic Index indicated that treatment with Co(NO_3_)_2_ reduced the MI by 46.6% to 52.2% compared to the water control, depending on the concentration used. Additionally, significant damage to epidermal cells and nuclei was observed in the groups treated with cobalt. Furthermore, the accumulation of Co(NO_3_)_2_ in the roots was notably higher in the Co1 and Co2 groups compared to the water control (Topuz and Uslu 2025).

### Protein and membrane damage

Heavy metals bind to the sulfhydryl groups in proteins, leading to protein misfolding and enzyme inactivation. They can also disrupt lipid bilayers, increasing membrane permeability and causing electrolyte leakage (Gupta et al. 2022). The loss of membrane integrity contributes to impaired transport and signaling within the cell. Exposure to 3 mM lead notably inhibited growth and chlorophyll levels, while elevating reactive oxygen species, lipid peroxidation, and membrane permeability (Saeed et al. 2025). Lead ion stress significantly increased the dry weight of seedlings, while all the tested heavy metals promoted the accumulation of soluble sugars. Although higher concentrations of heavy metals inhibited germination and growth of *Vigna radiata*. The adaptive strategies of V. radiata, including osmotic adjustment mediated by soluble sugars and enhanced biomass allocation, both of which contribute to its resilience under heavy metal stress (Qi et al. 2025).

## Plant defense mechanisms against heavy metals

Plants employ physical barriers, metabolic processes, and antioxidant systems for self-defense. These defense mechanisms can be broadly categorized into three main types: structural (physical) barriers, metabolic processes involving secondary metabolites, and intricate antioxidant systems that combat oxidative stress.

### Physical barriers

Plants establish structural barriers as the first line of defense against pathogen invasion and environmental stressors. These barriers include:

- *Cuticle and Waxes* The outermost layer of the epidermis is composed of cutin and waxes, which form a hydrophobic barrier that restricts pathogen entry and minimizes water loss (Serrano et al. 2014).
- *Cell Walls* Made up of cellulose, hemicellulose, pectin, and lignin, cell walls serve as a rigid defense structure (Lu et al. 2025). The cell wall serves as a passive barrier by adsorbing and immobilizing heavy metal ions through interactions with carboxyl, hydroxyl, and phosphate groups found in pectins, cellulose, and lignin. When exposed to metals such as Cd, the cell walls may thicken, leading to increased deposition of lignin and suberin. This process reduces the mobility of these metals. For example, cadmium can become trapped within the cell wall matrix, which helps limit its translocation to sensitive cellular components (Zhang et al. 2023).
- *Trichomes and Spines* These external features deter herbivores and provide mechanical defense (Le Gall et al. 2021). Trichomes accumulate heavy metals or produce secondary metabolites that mitigate metal toxicity (Riyazuddin et al. 2022).

### Metabolic defense mechanisms

When plants sense stress or pathogen invasion, they activate metabolic pathways that produce a wide variety of defensive compounds:

- *Phytoalexins* This involves the production of low-molecular-weight proteins, metallochaperones, and chelators such as Nicotianamine, putrescine, spermine, glutathione, phytochelatins, metallothioneins, organic acids, mugineic acids, and secondary metabolites including flavonoids and phenolic compounds. Plants release salicylic acid, jasmonic acid, and ethylene to mitigate stress (Agarwal et al. 2022).
- *Pathogenesis-Related (PR) Proteins* PR proteins, such as β-1,3-glucanases and chitinases, help break down fungal cell walls (van Loon et al. 2020).
- *Secondary Metabolites* Compounds like alkaloids, flavonoids, and terpenoids play significant roles in deterring herbivores and pathogens (Kessler and Kalske 2018).

### Antioxidant Defense Systems

ROS are important signaling molecules, but they can cause oxidative damage if not carefully regulated. Plants utilize both enzymatic and non-enzymatic antioxidants to manage ROS levels:

- *Enzymatic Antioxidants* These include SOD, CAT, and APX, which help eliminate harmful ROS, such as superoxide radicals and hydrogen peroxide (Roy et al. 2024).
-*Non-Enzymatic Antioxidants* Compounds like ascorbic acid, glutathione, tocopherols, and phenolic acids buffer ROS accumulation and assist in maintaining redox homeostasis (Noctor et al. 2023). These antioxidant systems are intricately linked with signaling pathways involving salicylic acid (SA), jasmonic acid (JA), and ABA, enabling plants to finely tune their defense responses based on the type of stress encountered (Roy et al. 2024).

Jorjani et al. (2024) observed that barley exhibited initial indications of metal toxicity akin to water scarcity, with genes associated with dehydration stress being overexpressed in response to Cd and Hg exposure. Chen et al. (2024a, b) identified oxidative stress and glutathione depletion in alfalfa roots as initial indicators of sensing and signal transduction after exposure to heavy metals. Through these mechanisms, plants can manage or detoxify heavy metals, making them key players in phytoremediation strategies aimed at mitigating heavy metal pollution. By exploiting or upregulating these defense mechanisms, plants can tolerate or even thrive in contaminated environments, contributing to bioremediation processes.

## Mode of action of toxic hms in plant cells

The presence of heavy metals in plants is indicated by the excessive accumulation of these elements within the cells, demonstrating their toxicity. HMs are classified into two categories: Fe, Cu, Cr, and Co were classified as redox active metals, while Cd, Zn, Ni, and Al were categorized as redox inactive metals (). ROS, including superoxide, hydrogen peroxide, and hydroxyl radicals, are generated through Haber–Weiss and Fenton reactions within cells. These reactions involve the interaction of iron and hydrogen peroxide, leading to the formation of highly reactive species that can cause cellular damage (Kessler et al. 2022). In contrast, redox inactive heavy metals contribute to oxidative stress through modifications of antioxidant redox defenses, changes in the electron transport chain, or the induction of lipid peroxidation (Al Mahmud et al. 2019). The latter HM may induce a response characterized by increased lipoxygenase (LOX) activity. In addition to this, HM also mediates HM toxicity through another significant mechanism characterized by a strong binding affinity with oxygen, nitrogen, and sulphur atoms (Shafi et al. 2024). This property allows HMs to inactivate enzymes through mechanisms such as protein misfolding, inhibition of enzyme activity, or disruption of redox homeostasis. The binding of Cd to sulfhydryl groups in structural proteins and enzymes results in damage to their functional properties (Rai et al. 2021).

**Fig. 4:**
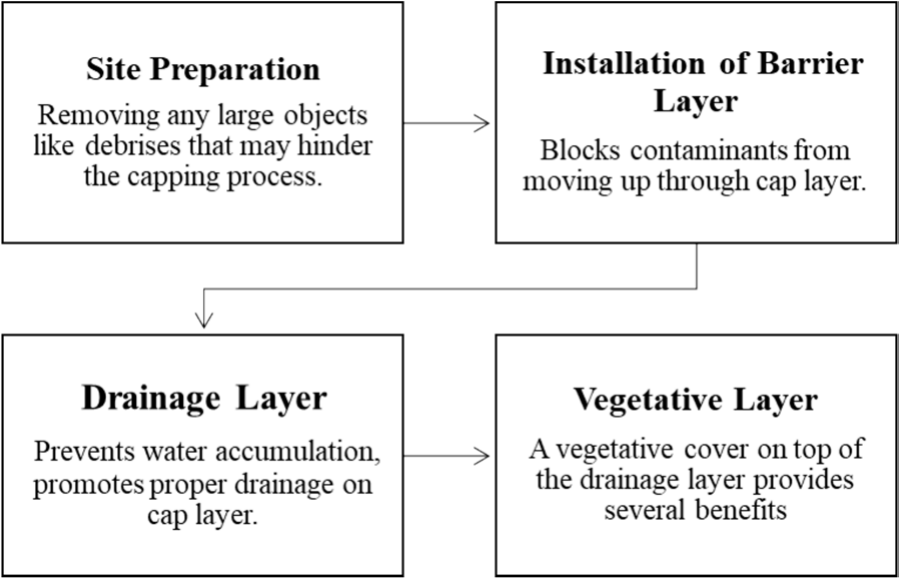
An overview of surface capping in remediating heavy metals

Heavy metals not only impact intact proteins but also modify the functionality of enzymes that rely on cofactors, which include metal ions (Fe^2+^, Mg^2+^, Cu^2+^, etc.) and organic molecules such as heme, biotin, FAD, NAD, and coenzyme A as shown in Fig. 4. The replacement of critical metal ions with harmful heavy metals leads to a decrease in enzyme activity. Divalent cations, including Co^2+^, Ni^2+^, and Zn^2+^, have the capacity to replace Mg^2+^ in ribulose 1,5-bisphosphate carboxylase/oxygenase (RuBisCO), leading to its inactivation (Aponte et al. 2020). When Cd^2+^ substitutes for Ca^2+^ in calmodulin, an essential signaling protein, it inhibits the activity of calmodulin-dependent phosphodiesterase (Li et al. 2022a, b). Heavy metals have the potential to compromise membrane integrity via multiple mechanisms. These include the induction of oxidation, cross-linking of proteins, obstruction of essential membrane proteins like H + -ATPase, and alterations to the composition and fluidity of membrane lipids (Wu et al. 2021). Furthermore, the elevation of HM stress results in increased concentrations of methylglyoxal (MG), a compound recognized for its cytotoxic characteristics. The glyoxalase system presents issues, and a reduction in glutathione (GSH) seems to be contributing factors to this increase, thereby exacerbating oxidative stress (Kanwal et al. 2024). In summary, the toxicity of heavy metals can be ascribed to three main factors: Auto oxidation, Fenton reactions, and the disruption of antioxidant systems, including oxido reductase and glyoxalase, lead to an increased production of both ROS and methylglyoxal (MG). Direct interactions with cellular proteins can lead to impairments in structural, catalytic, and transport functions, resulting from affinities for thiol, histidyl, and carboxyl groups. Functional failures arise when essential metal ions are replaced at critical binding sites.

## Cleanup of heavy metal-contaminated soils

The presence of heavy metals in soils represents a widespread concern that presents considerable hazards to human health and the integrity of food production systems. Although certain contamination originates from natural geological sources, the main contributors are anthropogenic activities. The activities encompass mining, smelting, military operations, electronic manufacturing, fossil fuel utilization, waste disposal, agrochemical applications, and irrigation practices. Coal, a commonly utilized fossil fuel, is comprised of heavy metals including Hg, Pb, Cd, Cr, Cu, Co, Zn, and Ni, with concentrations varying from 0.1 to 18 mg kg⁻^1^. Coal combustion results in the release of metals into the environment via vapor emissions, flue gas particulates, fly ash, and bottom ash (Tang et al. 2024).

Improper disposal of mine spoils, industrial waste, and construction debris significantly exacerbates soil contamination issues (Liu et al. 2018). It is important to recognize that certain agricultural practices significantly influence the accumulation of heavy metals in the soil. The use of phosphorus fertilizers, copper-based pesticides, biosolids, and animal manure, along with the irrigation practices involving sewage or inadequately treated industrial wastewater, significantly contributes to this problem (Adepoju et al. 2024). Globally, more than 5 million locations spanning approximately 20 million hectares are grappling with contamination from heavy metals and metalloids (Tauqeer et al. 2021). In China, over 1 million square kilometers, approximately 100 million hectares, are affected by heavy metal pollution (He et al. 2023).

Heavy metals in soils can significantly disrupt natural ecosystem services and present risks to human health as they enter the food chain (Tripathi et al. 2023). This issue can be approached through various methods, which can be categorized into two primary types: in-situ methods and ex-situ methods. The methods address the challenge of heavy metal-contaminated soils and encompass techniques such as surface capping, soil flushing, electrokinetic extraction, solidification, vitrification, and phytoremediation. Typically, there are five primary categories of remediation techniques: physical, chemical, electrical, thermal, and biological. Conversely, they can be categorized into three primary functional divisions: Methods focused on containment, such as capping or encapsulation, are employed to isolate contaminants. Methods based on transformation: such as stabilization or immobilization to reduce the bioavailability of heavy metals. Transport-based methods encompass processes such as extraction or removal to eliminate contaminants from the site (ur Rehman et al. 2023). These methods offer a range of solutions for addressing heavy metal contamination, tailored to the distinct characteristics of the affected area and the particular contaminants involved.

### In-situ remediation techniques for heavy metal-contaminated soils

In-situ remediation entails the direct treatment of contaminated soils at the location of contamination, thereby negating the necessity for excavation and off-site transportation. This method reduces soil disturbance, lowers the risk of contaminant exposure for workers and surrounding communities, and may offer greater cost-effectiveness in comparison to ex-situ techniques. Site-specific factors, such as weather conditions, soil permeability, contamination depth, and the potential for deep leaching of chemicals, require thorough evaluation prior to implementation (Liu et al. 2018).

#### Surface capping

Surface capping entails the application of a waterproof material over the contaminated zone, establishing a stable and protective barrier. This cap functions as a robust barrier, effectively stopping surface water from penetrating and restricting the movement of soil contaminants into both surface and groundwater. Nonetheless, capped soils forfeit their inherent environmental roles, especially their capacity to foster plant growth. As a result, capped areas are frequently transformed for civil applications, including parking lots or sports fields (Liu et al. 2018). The selection of a capping system is distinctly dependent on the specific conditions of the contamination site and the goals set for remediation (Fig. 4). Capping systems may consist of either a single layer or multiple layers, utilizing materials such as clay, concrete, asphalt, or high-density polyethylene. For optimal effectiveness, the cap must demonstrate sufficient structural integrity and dynamic stability, extending 60–90 cm beyond the horizontal boundary of the contaminated site (Liu et al. 2018; Shakoor et al. 2021; Mohasin et al. 2022).

Furthermore, water control structures like ditches, dikes, and slopes are commonly incorporated to regulate runoff and drainage, thereby enhancing the protection of the cap. In multilayer systems, the addition of a topsoil layer above the impermeable cover facilitates revegetation, thereby improving environmental aesthetics and ecological value (ur Rehman et al. 2023). Surface capping is especially effective in regions characterized by elevated contamination levels (e.g., Igeo > 3). Nonetheless, its use is confined to relatively small areas (e.g., < 2000 m^2^), since larger-scale applications present considerable logistical and construction hurdles. It is essential to take into account the depth and seasonal fluctuations of the groundwater table, along with local hydrogeological characteristics like ponds and runoff patterns, to guarantee the long-term stability of the cap (Liu et al. 2018).

#### Encapsulation

**Fig. 5:**
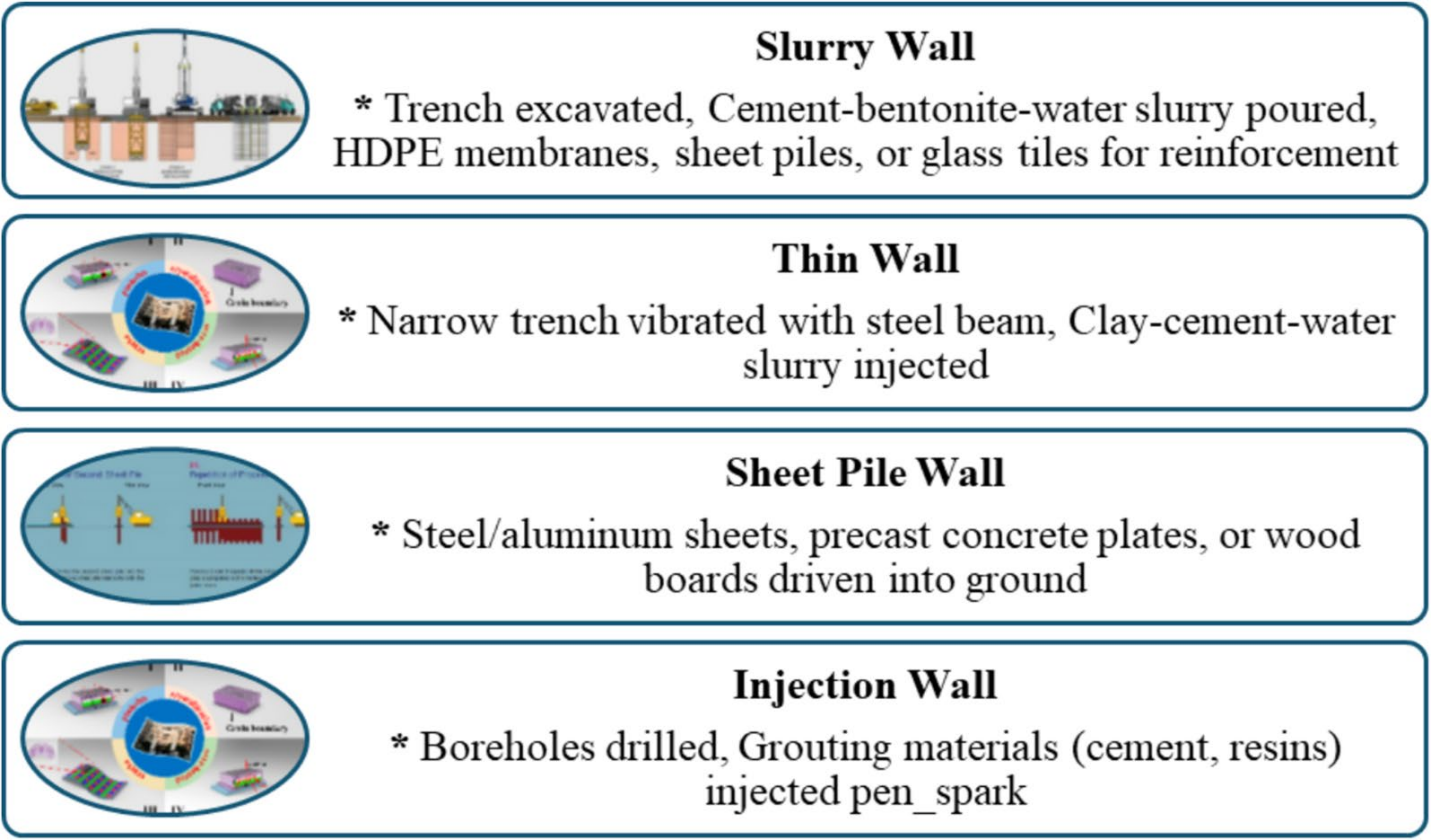
An overview of heavy metals remediation by encapsulation

Encapsulation, commonly known as a "barrier wall," "cutoff wall," or "liner" method, serves as a remediation strategy akin to surface capping. This process entails the immobilization and isolation of heavy metals in contaminated soils, thereby significantly minimizing their migration and the related environmental or health hazards. This method establishes a physical barrier system that includes low-permeability caps, underground barriers, and, on occasion, barrier floors. The contaminants are contained within the encapsulated area, preventing their dispersion and reducing exposure risks (Fig. 5). The low-permeability caps, typically constructed from synthetic textiles or clay layers, effectively minimize surface water infiltration, thereby inhibiting the leaching of contaminants into groundwater. In the interim, subterranean impermeable barriers impede the lateral migration of contaminants, thereby restricting diffusion and subsurface flow to adjacent regions.

Constructing subsurface vertical impermeable walls presents challenges when it comes to encapsulation. Slurry walls, thin walls, sheet pile walls, and injection walls represent various alternatives (Shakoor et al. 2021). Slurry Walls: Trench cutters or grab buckets are employed to excavate the trench to the appropriate depth. The cement-bentonite-water slurry is introduced into the trench, resulting in the formation of a wall measuring between 0.4 and 1 m. HDPE membranes, sheet piling, or glass tiles are incorporated into the slurry before hardening to enhance its strength and waterproofing properties. Sequential panel construction is excellent! It is crucial to ensure that watertight panel connections are maintained to uphold the integrity of wall strength. Fragile Boundaries: A pressure jet facilitates the vibration of a substantial steel beam into the ground to create a small excavation. Introducing a mixture of clay, cement, and water into the trench results in the formation of a 0.15-m wall. Soil compaction occurring during trench preparation leads to a decrease in permeability. Partitions of fabric: Impermeable walls can be constructed using materials such as steel or aluminum sheets, precast concrete panels, or timber boards that are driven into the ground. Interlocking mechanisms between adjacent piles provide stability and prevent water intrusion. In the context of injection walls, rotary casings are utilized to drill boreholes to the specified depth. High-pressure grouting materials such as cement suspension or artificial resins are injected. The grouting slurry effectively fills the gaps in boreholes and solidifies to form a "soilcrete" wall (Yenginar et al. 2024). Barrier walls are required to adhere to specific standards to function effectively. For example, it is essential to maintain hydraulic conductivity below 10⁻⁷ cm/s to regulate water movement. Minimal shrinkage is essential for maintaining structural integrity. Robust enough to address environmental challenges. Designed for sustained performance over an extended period (Swamynaidu et al. 2024). Encapsulation effectively retains contaminants, particularly in highly polluted areas. It effectively contains detrimental substances, preventing their release into the surrounding ecosystem.

#### Electrokinetic extraction

**Fig. 6:**
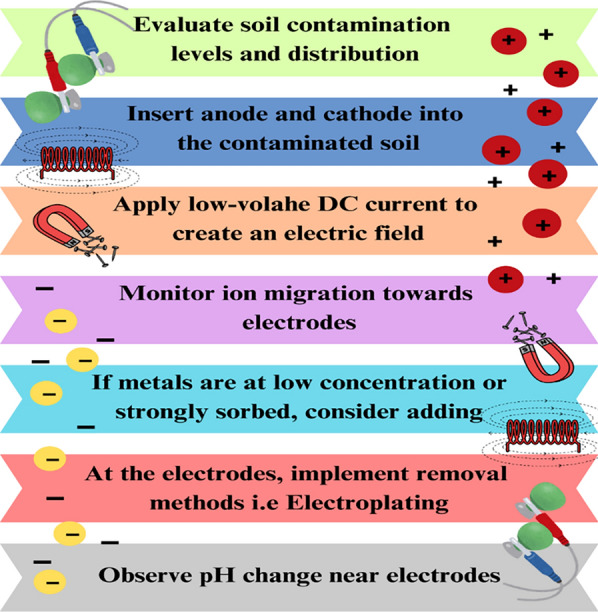
Heavy metal removal through electrokinetic extraction (EKE)

Electrokinetic extraction represents an innovative method for soil remediation, utilizing an electric field to facilitate the removal of heavy metals. Low-density direct current (DC) is applied through electrodes implanted in the soil. Cations migrate towards the cathode, while anions move towards the anode within the electric field. Electroplating, (co-)precipitation, solution pumping, and ion exchange resin complexation are methods for the removal of impurities from electrodes (Fig. 6; Mandal et al. 2023). Desorption, dissolution, and transport processes. Heavy metals are either desorbed or dissolved in soil solutions. Enhancement fluids are chemical additives that facilitate the mobility of sorbed metals. According to Sivapullaiah et al. (2015) and Song et al. (2016), EDTA, EDDS, DTPA, NTA, acetic acid, citric acid, and potassium iodide (KI) exhibit diverse effects on heavy metals in soil. Oxidation of water at the anode results in the formation of an acidic environment characterized by a high redox potential. The reduction of water at the cathode generates a basic environment characterized by a low redox potential. These reactions yield two chemical fronts: An oxidizing acid front is present near the anode. A decreasing base front is located near the cathode.

The acid front facilitates the movement of heavy metal ions by inhibiting the formation of metal hydroxides and carbonates. Soils exhibiting high buffering capacity can help neutralize acidity, thereby decreasing metal migration. Metal ions appear to accumulate at the front of the cathode base, potentially affecting the efficiency of transport to the electrode (Cheng et al. 2024). The pH, redox potential, electrical conductivity, and solution chemistry gradients differ among electrodes, resulting in a complex and dynamic system (Niri et al. 2025). Transporting metal ions in dilute soil solutions necessitates increased energy expenditure. The soil's capacity to buffer may help prevent acidification in its location. Cathode precipitation refers to the process where metal ions are reduced and deposited onto the cathode during electrochemical reactions. Heavy metals may precipitate prior to reaching the electrode, thereby diminishing the efficiency of their recovery. Electrokinetic extraction effectively targets contaminants at their source, rendering it a suitable method for remediating heavy metal pollution in soil. Comprehending the chemical and physical complexities of soil is essential for ensuring proper functionality.

#### Soil flushing

Soil flushing is a method of in-situ remediation aimed at eliminating heavy metal contaminants from soil. This process involves the passage of an extraction fluid through the soil, which helps to mobilize and remove the pollutants. The contaminated fluid is subsequently recovered, treated, and either reused or disposed of, rendering this method especially effective for homogenous, coarse-textured soils with high permeability. The extraction fluid is introduced into the soil via wells or permitted to permeate from the surface. The selection of fluid is essential for the effectiveness of the process, as it has a direct influence on the efficiency of contaminant mobilization. Chelating agents such as EDTA are frequently utilized due to their proven effectiveness in eliminating heavy metals. Research indicates that EDTA is more effective than alternatives like citric acid and tartaric acid, especially in the mobilization of metals such as copper, nickel, zinc, cadmium, and lead. In experiments with fortified loamy sand, EDTA demonstrated a superior extraction rate compared to citric or tartaric acids, with the efficiency of metal mobilization ranked as follows: Cu > Ni > Zn > Cd > Pb. Biodegradable chelating agents, including chitosan, have undergone testing and demonstrated potential in specific contexts. In a particular investigation, chitosan showed more effective effectiveness compared to EDTA in the extraction of copper and nickel from clay loam, achieving removal efficiencies of up to 75% for lead and 25% for copper under optimal conditions. The benefits of soil flushing are rooted in its economical nature and versatility. The extraction fluid is amenable to treatment and reuse, leading to decreased treatment costs. It is especially effective in soils characterized by high permeability, facilitating fluid movement and the recovery of contaminants. Nonetheless, this technique presents certain challenges. The effectiveness is constrained in fine-textured or low-permeability soils, and the removal efficiency fluctuates based on the particular metals and soil conditions. Furthermore, there exists a potential risk of contaminant dissemination if the process is not meticulously managed. In light of these challenges, soil flushing continues to be an effective and important method for addressing heavy metal-contaminated soils, provided that site characteristics and fluid formulations are meticulously optimized.

#### Chemical immobilization

In-situ chemical immobilization, commonly referred to as S/S, is a remediation technique that encapsulates contaminants within polluted soil. Chemicals play a crucial role in hardening soil or transforming mobile pollutant components, such as soluble and exchangeable forms, into precipitates or entities that are highly sorbed. Chemical immobilization does not remove dirt in the same way that extraction does. Rather, it significantly decreases the mobility, solubility, and concentrations of heavy metals in soil pore water, thereby minimizing their transfer to plants, microbes, and water (Hamid et al. 2019). In-situ and ex-situ solidification represent methods of chemical immobilization. Contaminated soil is treated by mixing it with in-situ binding agents such as cement, asphalt, fly ash, or clay through auger spin mixing, resulting in a solid, impermeable block. Bonding slurries can be introduced into the subsurface and combined with waste using an injector head and a large mixer to address deep soil contaminants. The solid block effectively prevents water penetration, rendering the contaminants inaccessible. Nonetheless, natural weathering or mechanical disturbances can compromise the integrity of the solid block, leading to the remobilization of pollutants. The cemented site could potentially limit future land use (Mohasin 2022; Mondal et al. 2023).

The process of chemical immobilization, such as stabilization, effectively reduces the movement of pollutants while maintaining the soil flexibility. In "in-situ fixation," the introduction of precipitation reagents or stabilizing chemicals to contaminated soil facilitates physicochemical interactions that reduce the mobility of heavy metals. Carbonates such as lime, phosphates including bone meal, ammonium phosphate, apatite, and hydroxyapatite, alkaline agents like fly ash and calcium hydroxide, clay and iron-containing minerals such as bauxite, red mud, goethite, greensand, molecular sieves, palygorskite, silica gel, vermiculite, and zeolites, along with organic matter like chitosan, starch xanthate, peat, compost, manure, and activated carbon. In order to mitigate the movement of heavy metals and other pollutants in contaminated soils, in-situ chemical immobilization can be employed to solidify or stabilize these contaminants. The choice of appropriate materials, thorough site assessments, and ongoing monitoring to contain pollutants over time are crucial for ensuring long-term effectiveness and environmental safety (Tajudin et al. 2016).

### *Ex-situ* remediation techniques

Ex-situ remediation involves the removal of contaminated soil from its original site for treatment at a designated off-site facility, followed by proper disposal. Although this approach incurs higher costs due to excavation, transportation, and disposal processes, it offers better control over treatment conditions, allowing for accelerated and more effective remediation.

#### Landfilling

**Fig. 7:**
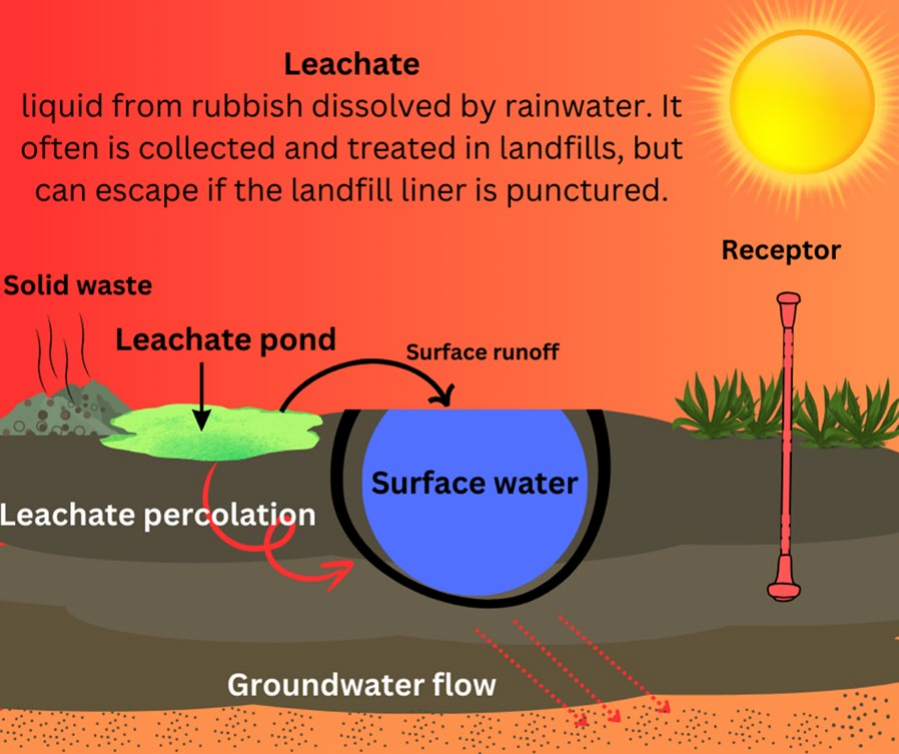
An overview of secure landfill structure

Landfilling, also known as "dig and haul," is one of the simplest forms of soil remediation. Contaminated soil is excavated and transported to secure landfill sites designed with engineered features like impermeable liners, leachate drains, and dike enclosures to prevent environmental contamination (Liu et al. 2018). While effective in isolating pollutants, landfilling does not degrade or neutralize contaminants and may lead to long-term environmental challenges. (Fig. 7).

#### Soil Washing

**Fig. 8:**
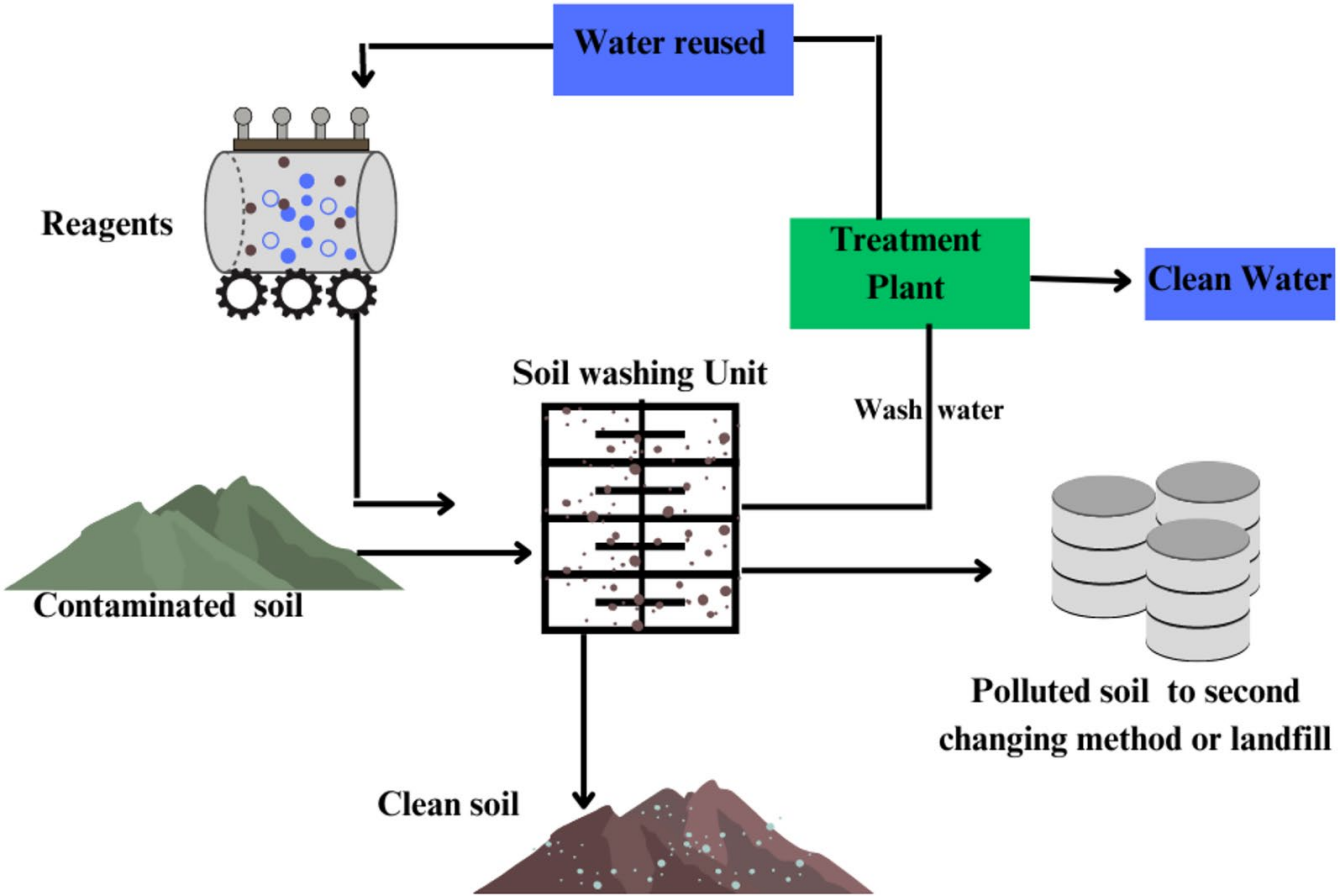
A typical soil washing procedure for removing heavy metal contaminants

Soil contaminated with heavy metals undergoes treatment through chemical and physical methods. Pollutants are eliminated by washing the soil ex-situ with targeted solutions (Santos et al. 2025). Following the excavation of contaminated soil, the material is subjected to crushing and screening processes to eliminate larger debris such as plastics, wood, and stones. Magnets attract and eliminate magnetic materials. Screened dirt (< 5 mm) is thoroughly mixed with a washing solution through sonication or mechanical agitation. Subsequently, the soil undergoes sieving or hydrocyclone treatment to eliminate coarse sand and gravel (> 0.05 mm) from the finer silt and clay (< 0.05 mm). Clean the coarse fraction, which has lower contamination, and return it. The washing solution facilitates the settling, rinsing, and return of silt and clay particles to the site. Rinsing and washing water undergoes processes of reuse, recycling, or is directed to a wastewater treatment facility for proper disposal. Waste sludge generated from wastewater treatment undergoes a process of solidification or stabilization prior to its transfer to landfills. This procedure involves the reuse and treatment of waste to mitigate soil heavy metal contamination and lessen environmental impact (Fig. 8).

#### Solidification

Ex-situ soil solidification entails the excavation of metal-contaminated soil, followed by screening at a treatment facility to eliminate coarse debris larger than 5 cm. An extruder combines screened soil with a binding agent, which evenly distributes throughout the soil to create a water-resistant solid structure that encapsulates pollutants. This technique is referred to as “microencapsulation.” Ex-situ stabilization involves the chemical immobilization of pollutants through the application of a stabilizing agent rather than a binding substance (Liu et al. 2018). Molten bitumen, emulsified asphalt, modified sulfur cement (a thermoplastic with a melting temperature of 127–149 °C), polyethylene, pozzolan cement (fly ash, kiln dust, pumice, or blast furnace slag), and Portland cement serve to bind contaminants. Soluble phosphate or lime can effectively immobilize heavy metals in soil while maintaining its pliability.

#### Vitrification

Vitrification transforms contaminated soil into glass-like materials through the application of heat. Vitrification, established and validated since the 1980s, generates a high-temperature zone (> 1500 °C) in contaminated soil through the application of intensive energy. The soil transforms into "lava," which solidifies into glass in this area. Organic pollutants are removed, and heavy metals are encapsulated within the glass matrix (Perveen et al. 2024). This establishes a strong, long-lasting, chemically stable, and leaching-resistant structure. The three primary techniques for vitrification include electrical methods, which involve applying high voltage electricity to graphite electrodes placed at the contamination site; thermal methods, which utilize microwave radiation or natural gas to heat contaminated soil within a rotary retort; and plasma methods, which rely on electrical discharge to create gas plasma. Vitrification is not advisable for soils characterized by elevated organic matter levels exceeding 7%, moisture content surpassing 10%, or the presence of volatile or flammable organics. To achieve the best outcomes, soil should have a composition of 2–5% monovalent alkaline cations (Na^+^ and K^+^). Vitrification results in a robust, leaching-resistant, glass-like material; however, it may undergo gradual de-vitrification due to weathering during field storage. The dissolution and leaching of chemical components in silicate glasses can occur over thousands of years, with variations ranging from 0.1% to 25%, depending on the species (Conte et al. 2024).

## Bioremediation: an environmentally resilient strategy for the problem of heavy metal pollution

Bioremediation effectively restores soil, water, and air quality while ensuring environmental safety. This approach employs microorganisms and plants to transform hazardous substances into less harmful or non-toxic variants. These organisms metabolize contaminants, converting them into water, carbon dioxide, and biomass. Bioremediation can be conducted either in-situ or ex-situ, contingent upon the type of pollution, the specific pollutants involved, and the conditions of the site. This method is primarily employed to remove organic contaminants from soil and groundwater (Alori et al. 2022). Microorganisms possess the ability to detoxify heavy metals using a range of mechanisms, including valence transformation, biosorption, chemical precipitation, and volatilization into less harmful gaseous forms (Alori et al. 2022).

Bioremediation is often employed in conjunction with soil flushing and phytoextraction to solubilize heavy metals and eliminate them from contaminated soils. In calcareous soils, the iron-reducing bacteria *Desulfuromonas palmitatis* enhances the release of arsenic (Liu et al. 2018). The role of microbial activity is essential in the process of bioremediation. *Bacillus subtilis* and *Torulopsis bombicola* produce biosurfactants such as surfactin, rhamnolipids, sophorolipids, aescin, and saponin, which assist in the solubilization of metals in soils (Chernyshova et al. 2023). In phyto-assisted bioremediation, microorganisms associated with the rhizosphere enhance plant growth and resilience in environments contaminated with heavy metals (Mishra et al. 2017). Bioremediation has the potential to volatilize mercury in situ through the action of microbes. Bacteria transform methylmercury into Hg(II), which is subsequently reduced to elemental mercury (Hg⁰) and emitted into the atmosphere, necessitating rigorous monitoring to avert secondary contamination (Mahbub et al. 2017).

### Types of bioremediations

#### Microbial bioremediation

**Fig. 9:**
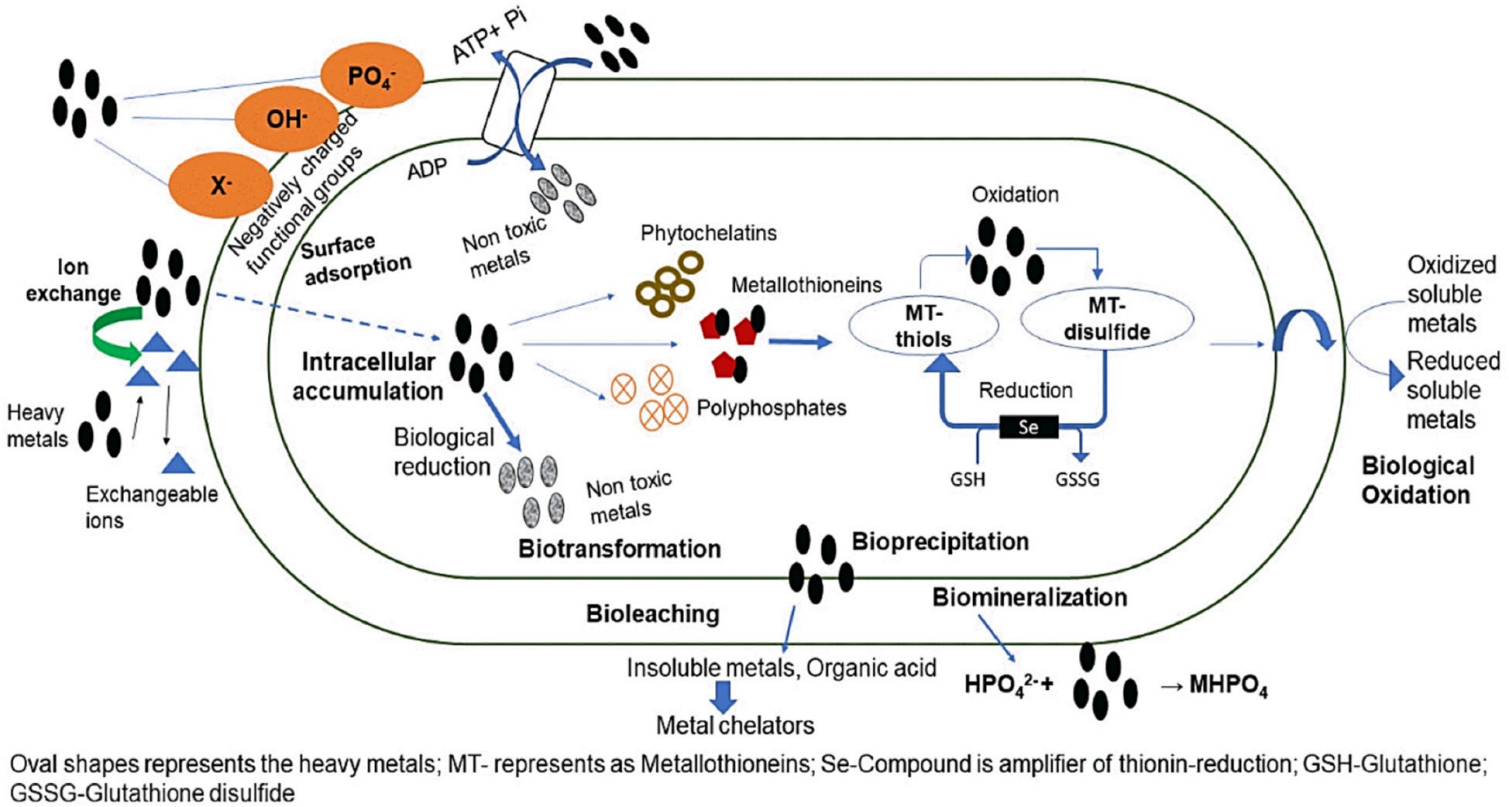
Various mechanisms of microbial remediation of HM-contaminated soil (Jeyakumar et al. 2023)

Microbial bioremediation employs bacteria and fungi to degrade organic contaminants as shown in Fig. 9. Microorganisms could have originated from the contaminated site or another location. Inoculants such as yeast, mycorrhizae, cyanobacteria, and rhizobacteria have been proposed to enhance plant performance, fitness, tolerance, growth, and phytoremediation (Raklami et al. 2023). To remediate soil contaminated with heavy metals, microorganisms need to possess various mechanisms to defend against metal toxicity (Table 2). The processes encompass volatilization, enzymatic detoxification, precipitation, metal chelator production, as well as extracellular and intracellular sequestration (Ojuederie and Babalola 2017). The negatively charged anionic structures of microbial cell walls facilitate the attachment of metals through covalent bonding, ionic interactions, or Van der Waals forces, contributing to the remediation of polluted media (Raklami et al. 2022). The presence of functional groups like carboxyl, ester, sulfhydryl, sulfonate, thioether, phosphoryl, amine, hydroxyl, and thiol has been reported in these toxic sites (Mishra et al. 2017). Furthermore, microbial communities generate extracellular polymeric substances (EPS) that consist of polysaccharides, glycoproteins, lipopolysaccharides, and soluble peptides. The EPS components, which encompass nucleic acids, lipids, proteins, and complex carbohydrates, provide a significant array of metal-binding sites—such as carboxyl, hydroxyl, amino, sulfhydryl, and phosphate groups—facilitating the stabilization of heavy metals through biosorption (Yue et al. 2015).

**Table 2:** Role of microorganisms in phytoremediation of heavy metal

Microorganisms	PGP properties	Crop	Heavy metal	Effects	References
Bacteria					
*Streptomyces* sp.	IAA, ACCD, Zeatin, GA, P solubilization	*Zea mays*	As, Cr	Increased germination features included FW and DM, photosynthetic pigments, shoot and root tolerance indexes, leaf length, root, and cob	AL-huqail and El-bondkly (2022)
*Proteus* sp., *Pseudomonas* sp., *E. meliloti*	IAA, Biofilm, P solubilization, K solubilization	*M. sativa*	Cu, Zn, Pb	Inoculation reduced the amounts of antioxidant enzymes, enhanced growth, elevated physiological parameters, and reduced metal stress	Raklami et al. (2019)
*Streptomyces pactum*	n/s	*Sorghum bicolor*	Zn, Pb, Cd, Cu	The dry mass of the shoots and roots and chlorophyll content increased. Glucosidase, alkaline phosphatase, and urease activity in the soil were also enhanced, and antioxidant activity (POD, PAL, and PPO) in plants was reduced	Ali et al. (2017)
*Bacillus* sp.	IAA, hydrolytic and ligninolytic enzymes, Siderophores	*Phragmites communis*	Fe, Cu, Zn, Cd, Mn, Ni, Pb, As	In situ phytoremediation assisted by bacterial inoculation reduced HMs’ contents	Sharma et al. (2021)
*Acinetobacter lwoffii*	IAA, ACCD, EPS, siderophore, P solubilization	*Vigna radiata*	As	Enhanced chlorphyll and carotenoid content, growth, and number of plants per pot The inoculation reduced the generation of ROS	Das and Sarkar (2018)
*Aliinostoc* sp.	phosphatase production, N fixation	*Oryza sativa*	Cd	Enhanced rice yield and growth, immobilized Cd and reduced grain translocation and absorption Aliinostoc sp. binds with Cd by amino, carboxyl, and hydroxyl groups	Hu et al. (2022)
*Arthrobacter* sp., *Bacillus altitudinis, Bacillus megatherium, Sphingomonas* sp.	ACCD, IAA, Siderophore, P solubilization	*Brassica napus*	Cd	Increased shoot biomass production,	Pan et al. (2017)
*Bacillus cereus*	IAA, siderophores	*Zea mays*	Cd, Cu, Ni, Pb, Zn	Increased the biomass, pigments, phenols, protein, and antioxidants	Bruno et al. (2021)
*Klebsiella* sp.	IAA, EPS, NH_3_ P solubilization	*V. radiata*	Cd, Cu, Pb	Inoculation increased plant growth under HM stress	Chakraborty et al. (2021)
*P. indica*	n/s	*Cenchrus purpureus*	Cd	Inoculation promoted plants' accumulation of Cd and plant growth	Li et al. (2022b)
*Pseudomonas* sp.	IAA, EPS, HCN P solubilization, N fixation, siderophores	*M. sativa*	Cr	Improved dry mass of the shoots and roots, reduced stress indicators, raised Chl content and reduced the root's Cr content	Tirry et al. (2021)
*Bacillus* XZM	EPS	*Helianthus annuus*	As	Stimulated the growth, enhanced enzyme activities, and reduced As contents	Mao et al. (2024)
Cyanobacteria					
*Oscillatoria* sp.	n/s	*Portulaca oleracea*	Cr, Fe, Al, Zn	Cyanobacteria enhanced Plant growth, Chlorophyll a, and N content and reduced the extractable proportion of heavy metals and their accumulation inside plant tissues	Zanganeh et al. (2022)
*Oscillatoria* sp., *Leptolyngbya* sp.	n/s	*Lactuca sativa* and *Raphanus sativus*	Fe, As, Pb, Cr, Ni	Enhanced vigor index, root and hypocotyl lengths, and nutrient content as a result of lower HM bioavailability and high nutrient content	Atigh et al. (2020)
*Leptolyngbya* sp	EPS	*Helianthus annuus*	As	Increased the enzyme activities (catalase, sucrase, urease) in the rhizosphere soil of sunflower and reduced the toxic effect of As on plant	Mao et al. (2024)
Fungi and arbuscular mycorrhizal					
*Aspergillus flavus*	EPS	*Triticum aestivum*	Pb	Increased the growth and physiological characteristics, and decreased oxidative stress through decreasing (Catalase, peroxidase, and lipid peroxidation)	EL-khawaga et al. (2024)
*Aspergillus niger,* *Penicillium chrysosporium*	ACCD, IAA, Gibberellins P solubilization, siderophores	*V. faba*	Cd, Pb	Inoculated plants mitigated metal-induced oxidative stress by blocking metal transport and reducing electrolyte leakage and lipid peroxidation	El-Mahdy et al. (2021)
*Glomus mosseae, Sinorhizobium meliloti*	n/s	*M. sativa*	Cd	Increased alfalfa resilience to cadmium stress through increased activity of antioxidant enzymes	Wang et al. (2021)
*Debaryomyces hansenii*	IAA, P and Zn solubilization, Siderophores	*O. sativa*	As	Plant growth, total chlorophyll, sugar, and proline were all enhanced by yeast inoculation - By scavenging ROS, inoculation enhanced plant detoxification	Kaur et al. (2020)
*Bacillus* sp., *Pseudomonas* sp., *Glomus mosseae*	IAA, HCN, siderophores, P solubilization	*Eucalyptus camaldulensis*	Cd	Inoculation improved plant growth traits (shoot DM, height, and leaf area)	Motesharezadeh et al. (2017)
*Funneliformis mosseae*	n/s	*Glycine max*	Cu, Pb, Zn	AMF inoculum increased growth and seed yield	Adeyemi et al. (2021)
*F. mosseae, Diversispora spurcum*	n/s	*Cynodon dactylon*	Pb, Zn, Cd	Improved soil pH and P, S, and HMs’ uptake by Bermuda grass, and reduced available Pb and Zn in soils and Pb in shoots	Zhan et al. (2019)
*Glomus aggregatum,* *G. intraradices, Glomus elunicatum, Glomus versiforme*	n/s	*M. sativa*	Cd	AMF improved plant growth and contents of N and P in plant shoots and decreased Cd uptake in plant tissues	Zhang et al. (2019)
*Penicillium janthinellum*	IAA adsorption	*C. dactylon*	Cd	In the presence of Cd, inoculation promoted plant growth. Specifically, it enhanced Cd uptake in the stem and roots of Bermuda grass	Xie et al. (2021)
*Rhizophagus fasciculatus*, *Rhizophagus intraradices*, *F. mosseae G. aggregatum*	n/s	*Z. mays*	Cd, Cr, Ni, Pb, Fe, Zn, Cr, Mn	AMF strongly impacted the growth of plants and the capacity for phytoremediation, increased the shoot and root's proline, chlorophyll content, and P content - The activity of the soil enzymes (alkaline phosphatase, dehydrogenase, β-glucosidase, and acid) was enhanced by AMF inoculation	Singh et al. (2019)

*n/s* not specified. *ACCD* 1-Aminocyclopropane-1-carboxylic acid deaminase, *EPS* exopolysaccharides, *HCN* hydrogen cyanide, *IAA* Indole-3-acetic acid, *ROS* Reactive oxygen species, *AMF* arbuscular mycorrhizal fungi

Mycorrhizae play a crucial role in reducing metal absorption or enhancing bioavailability, which is vital for phytoremediation efforts. Nonetheless, the phytoremediation effects of mycorrhizae are significantly influenced by both ecotype and fungal species (Coninx et al. 2017). Gloaming effectively immobilizes metals in soil and establishes a physical barrier, thereby minimizing heavy metal absorption, transport, and bioaccumulation in plant tissues. The chelation and sequestration of metals within fungal hyphae serve as a crucial mechanism for protection (Riaz et al. 2021). The process of mycorrhizae-assisted phytoextraction encompasses the production of chelating agents, the transformation of metals, and the transfer of metals from fungal cells to plant cells. The interactions illustrate the capacity of mycorrhizae to bioremediate soils contaminated with heavy metals, thereby enhancing environmental sustainability (Cabral et al. 2015).

Abu-Elsaoud et al. (2017) found that by preventing Zn buildup in the wheat plant, inoculation with the native mycorrhizal fungus *Funneliformis geosporum* enhances soil quality and boosts wheat yields even in the presence of elevated zinc concentrations. Vaseem et al. (2017) found that *Pleurotus ostreatus* can effectively remove several heavy metals—including Zn, Cr, Pb, Cu, Co, and Ni—from coal washery effluents. The accumulation of these heavy metals led to an increase in metallothionein protein concentration, lipid peroxidation, and the activity of antioxidant enzymes in the mycelium, enhancing the fungus's tolerance to heavy metal toxicity. Additionally, various fungi such as *Rhizopus microsporus, Fomitopsis meliae, Trichoderma ghanense*, and *Absidia cylindrospora* can tolerate and biosorb As, Cu, Fe, Pb, and Cd (Albert et al. 2018; Oladipo et al. 2018). Cecchi et al. (2017) reported that *Trichoderma harzianum*, isolated from a silver-polluted site, was the most efficient at accumulating and tolerating silver. Indigenous fungi found in gold and silver sites, as well as gem mines, can sequester these metals, providing an economic advantage. Khan et al. (2019b) demonstrated that various metallotolerant *Aspergillus* species, isolated from industrial estate soil, showed high efficiency in removing Pb and Hg. Furthermore, *Penicillium rubens* and *Aspergillus* species from the same location were capable of sequestering Cd and Cr (Khan et al. 2019a).

*Trichoderma species* and *Aspergillus niger* demonstrated greater removal rates than bacteria-mediated remediation and an eightfold increase in As removal compared to traditional chemical treatments. Fungal-induced bioleaching was used to eliminate non-mobile Zn and Cd (Dell'anno et al. 2022). *Aspergillus sydowii*, removed 26% of the Cr(VI) by intracellular deposition of Cr_2_O_3_ and exopolysaccharide-mediated processes. Finally, despite the lack of a defined mechanism for immobilization, *Kalmusia italica*, a member of the Kalmusia genus, isolated from maritime sediments, has demonstrated tolerance to Ni, Cr, Pb, and Zn (Sumathi et al. 2020).

The effectiveness of *Bacillus zhejiangensis* CEIB S4-3 in eliminating almost 90% of Cd^2⁺^ and 91% of Pb^2⁺^ (50 mg/L) when tested separately was noted by Hernández-Guerrero et al. (2025). The clearance rates for Cd and Pb in a Cd–Pb mixture, however, decreased to 59% and 75%, respectively. Pb was mostly eliminated by intracellular bioaccumulation and Cd by external biosorption. Heavy metal resistance-related genes, such as those pertaining to sensing, transcriptional responses, and efflux systems, were found by genome analysis. Furthermore, strains BRS 11 and BRS 17 of *Bacillus cereus* shown a considerable synergistic resistance and a high tolerance to heavy metals, efficiently reducing Cr(VI) and Ni(II) with electron donors (Dutta et al. 2021). *Pseudoxanthomonas mexicana* GTZY, a wastewater bacterium that can convert arsenic and mercury, is another noteworthy bioremediator. According to Abdul Raheem et al. (2024), merA gene amplification verified that GTZY eliminated 84.3% of mercury through Hg(II) volatilization. Furthermore, it effectively eliminated 68.5% of arsenate and 63.2% of arsenite, with resistance values above 175 mM and 55 mM, respectively.

In comparison to monocultures, native bacterial consortia consisting of *Shewanella putrefaciens, Aeromonas hydrophila*, and *Aeromonas caviae* showed better resistance and removal efficiency for Cu (47.02%), Ni (61.49%), and Zn (61.93%) in heavy metal bioremediation. These bacteria were perfect candidates for heavy metal remediation because of their synergistic interactions, which allowed for several metal removal methods, such as bioaccumulation, biosorption, and biotransformation (Khidr et al. 2025). *Lysinbacillus fusiformis* and *Bacillus tropicus* worked together to remove 84.2% of Cr(VI) and 90.3% of lubricating oil in just six days. Bacteria that were exposed to chromium secreted extracellular proteins that helped with metal detoxification and the breakdown of organic pollutants (Huang et al. 2025a, b).

##### Mechanisms of microbial bioremediation

Microorganisms interact with pollutants through several biochemical and physiological processes, which may vary depending on the type of pollutant and environmental conditions.

**Biosorption**

Microbial cells, especially their cell walls and extracellular polymeric substances (EPS), passively adsorb heavy metals and organic pollutants. Bacteria produce iron-chelating substances known as siderophores, which enhance the mobility of metals and reduce their bioavailability, facilitating their removal from soil. Sulfate-reducing bacteria, such as *Desulfovibrio desulfuricans*, can convert sulfate into hydrogen sulfide. This hydrogen sulfide then reacts with heavy metals like Cd and Zn to form insoluble metal sulfides (Chibuike and Obiora 2014)*.*

**Fig. 10:**
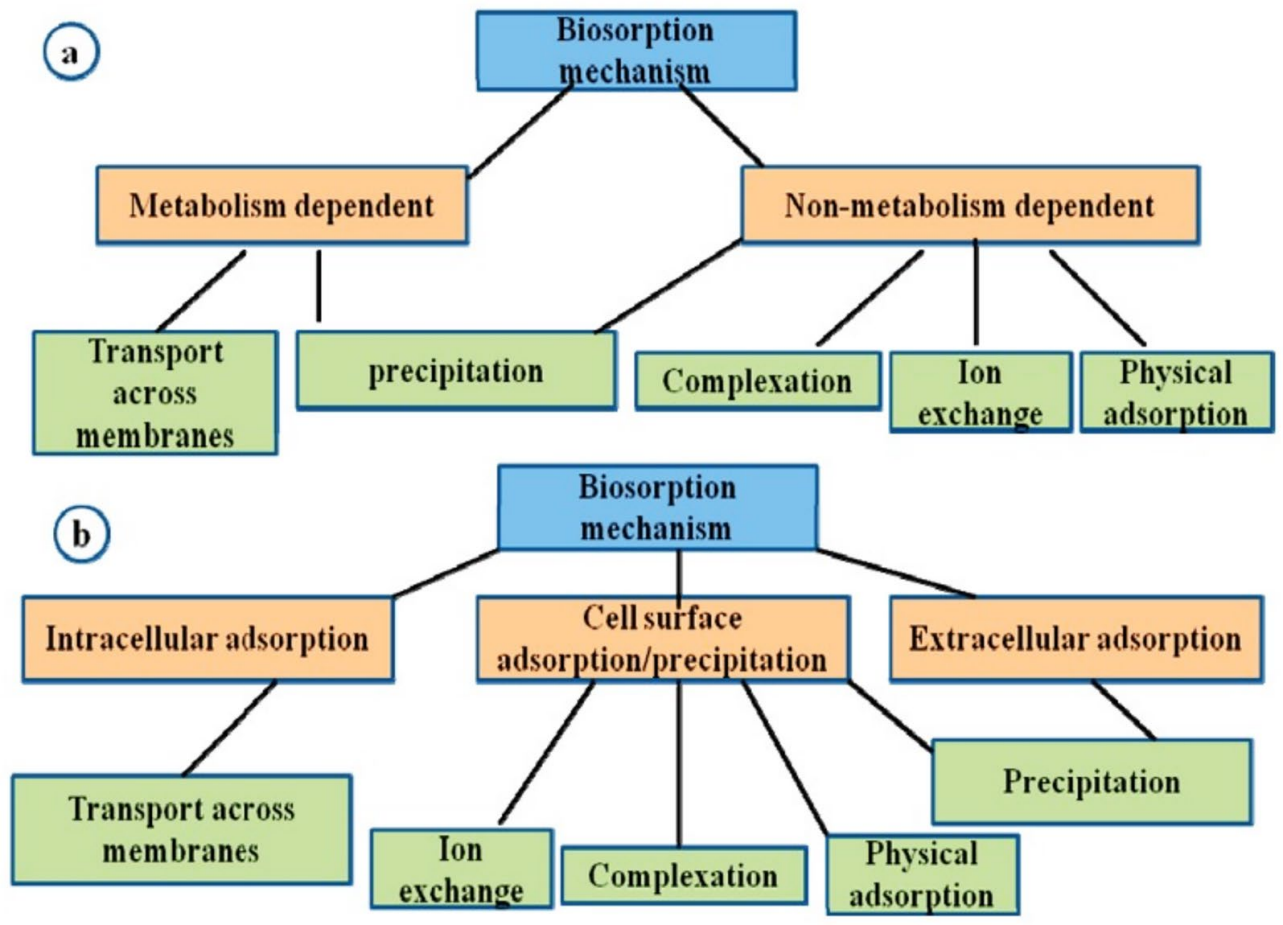
Mechanisms of biosorption depend on (**a**) cell metabolism and (**b**) the specific locations within the cell where metals are removed (Ndeddy and Babalola 2016)

Biosorption refers to the process of removing heavy metals, compounds, and particulates from a solution using low-cost biological materials, such as dead biomass or natural materials with strong degradative abilities (Srivastava and Dwivedi 2015). The mechanisms involved in biosorption can either depend on the cell's metabolism or occur independently of it. These mechanisms may include extracellular accumulation and precipitation, cell surface sorption and precipitation, and intracellular accumulation (Beiyuan et al. 2017)*.* These processes are illustrated in Fig. 10.

The biosorptive abilities of microbial biomass can vary significantly between different groups. The effectiveness of each biosorbent is influenced by its previous history and any pretreatment it undergoes, as well as the specific experimental conditions applied (Ayangbenro and Babalol 2017; Ghanaim et al. 2025a)*.* Negatively charged functional groups found in the biomolecules of microbial cell walls, such as hydroxyl, phosphate, and carbonyl groups, readily bind to heavy metal ions (Dixit et al. 2015). Additionally, bacterial functional groups, including the uronic acid in carboxyl groups (RCOO −) and sulfate groups (SO_4_^2−^), can facilitate ion exchange. The cell walls of Gram-positive bacteria are composed of peptidoglycan layers that contain amino acids such as alanine and glutamic acid, along with meso-diaminopimelic acid and teichoic acid (Ojuederie and Babalola 2017). In contrast, Gram-negative bacteria have cell walls that include enzymes, glycoproteins, lipopolysaccharides, lipoproteins, and phospholipids (Ayangbenro and Babalol 2017). The components of the cell wall serve as active sites for binding processes in bacteria, acting as ligands for the attachment of metal ions. This function plays a crucial role in the remediation of contaminated environments. Bacteria are effective biosorbents for treating polluted areas because they can grow under controlled conditions and tolerate extreme environmental factors. They excel at removing heavy metals from contaminated settings (Ojuederie and Babalola 2017).

Similarly, fungi can resist and detoxify metal ions through active accumulation, intracellular and extracellular precipitation, and valence transformation. As a result, they are considered potential biocatalysts for the bioremediation of heavy metals, as they can absorb these metals into their mycelium and spores (El-Mahdy et al. 2021). Yeast, specifically *Saccharomyces cerevisiae*, also serves as an effective agent for bioremediation. This is primarily due to their capability to remove toxic metals from contaminated wastewaters through a process called biosorption, which involves ion exchange (Qader et al. 2025).

Algae produce a large biomass, giving them a higher sorption capacity than many other microbial biosorbents. Mustapha and Halimoon (2015) reported a biosorption efficiency of 15.3–84.6% when using algae, which is significantly higher compared to other microbial options. This process also occurs through ion exchange mechanisms. Furthermore, it has been observed that brown marine algae effectively remediate heavy metals such as cadmium (Cd), nickel (Ni), and lead (Pb) due to the presence of various chemical groups on their surfaces, including carboxyl, sulfonate, amino, and sulfhydryl groups.

**Bioaccumulation**

Certain microbes actively uptake and accumulate pollutants, particularly heavy metals, within their cells for detoxification or storage. Example: *Spirulina platensis* accumulates nickel, copper and zinc ions intracellularly (Kaamoush et al. 2022). Microbes utilize heavy metals and trace elements as terminal electron acceptors to obtain energy necessary for detoxifying metals through both enzymatic and non-enzymatic processes. Bacterial cells can also bioaccumulate heavy metal ions, storing them in both particulate and insoluble forms, along with their by-products. A crucial component of these bacteria, which possess ion sequestration capabilities, is exopolysaccharide (EPS). Exopolysaccharide primarily consists of complex, high molecular weight organic macromolecules, such as polysaccharides, along with smaller amounts of proteins and uronic acids (Ghanaim et al. 2025b). EPS helps protect bacteria from environmental stresses like heavy metal toxicity, drought, and salinity. Certain microorganisms, including *Agrobacterium* spp., *Alcaligenes faecalis, Xanthomonas campestris, Bacillus spp., Zygomonas mobilis, Leuconostoc*, *Pseudomonas* spp., and *Acetobacter xylinum*, have been identified as producers of EPS (Revin et al. 2023).

To achieve heavy metal remediation through bacterial EPS, the focus should be on using the negatively charged EPS, which is rich in anionic functional groups, as a suitable biosorbent. Commercial bacterial EPS with the necessary anionic properties include alginate (*Pseudomonas aeruginosa* and *Azotobacter vinelandii*), gellan (*Sphingomonas paucimobilis*), hyaluronan (*Pseudomonas aeruginosa*, *Pasteurella multocida*, and attenuated strains of *Streptococci*), xanthan (*Xanthomonas campestris*), galactopol (*Pseudomonas oleovorans*), and fucopol (*Enterobacter* A47). The production of exopolysaccharide is closely linked to biofilm formation, which plays a vital role in the biosorption and biomineralization of metal ions (Gupta and Diwan 2016).

**Bio-mineralization**

Bio-mineralization is a process in which microbes facilitate the synthesis of minerals to address heavy metals (HMs). Different bacterial species contribute to the immobilization of Pb and Cr through carbon mineralization (He et al. 2019). For instance, the bacterial strain *Sporosarcina ginsengisoli* has been shown to immobilize various HMs through the biomineralization of calcite, aragonite, and vaterite (Tang et al. 2024).

Fungal species have also demonstrated promising results in bio-mineralization. For example, *Penicillium chrysogenum* facilitates the mineralization of Pb and Cr (Qian et al. 2017) and effectively promotes the bio-mineralization of Pb (Povedano-Priego et al. 2017). Additionally, *Bacillus subtilis* triggers the bio-mineralization of Pb through the synthesis of phosphate (PO_4_^3−^) released during Pb breakdown (Lin et al. 2016). Moreover, other studies have reported that *Pseudomonas putida* forms carbonate and phosphate minerals, which accelerate the precipitation of Cd (Li et al. 2016).

Microbes are critical to the bio-mineralization process as they produce mineral deposits that help immobilize HMs. These microbes generate extracellular polymeric substances (EPS), specific metabolites, and organic acids that promote the formation of mineral deposits, leading to the immobilization of HMs (Qian et al. 2017). Siderophores and polysaccharides produced by microbes bind to HMs, forming complexes that reduce metal uptake and accumulation in plants. Furthermore, these compounds facilitate the sequestration of metals in the soil, thereby lowering the toxicity of metals for plants.

**Biotransformation**

Microorganisms enzymatically convert pollutants to less toxic or inert forms through redox reactions or hydrolysis. Biotransformation is the process by which a chemical compound is structurally altered, resulting in the synthesis of a more polar molecule (Pervaiz et al. 2013). In simpler terms, when metals interact with microorganisms, toxic metals and organic compounds can be converted into less hazardous forms. This mechanism allows microorganisms to adapt to environmental changes.

Microbial transformations can occur through various processes, including the formation of new carbon bonds, isomerization, the introduction of functional groups, oxidation, reduction, condensation, hydrolysis, methylation, and demethylation (Pande et al. 2022). Research has documented the transformation of metals through microbial activity. For instance, Thatoi et al. (2014) reported that the Cr(VI)-tolerant bacterium Bacillus sp. SFC 500-1E can reduce the hazardous chromium(VI) to the less toxic chromium(III) using NADH-dependent reductase.

**Bioprecipitation**

Microbial bioprecipitation is an innovative bioremediation technique that utilizes microbes to transform soluble heavy metal ions into insoluble precipitates, effectively removing them from water and soil environments. This method decreases the bioavailability and toxicity of metals without producing harmful by-products. It is particularly effective for metals such as Pb, Cd, Cu, Zn, and Cr. Some microbes induce the formation of insoluble metal compounds by altering pH or redox potential or producing precipitating agents (e.g., sulfides). Sulfate-reducing bacteria precipitate heavy metals as metal sulfides (Mafane et al. 2025).

**Bio-Volatilization**

Bio-volatilization is a process in which microbes enzymatically convert heavy metals (HMs) into volatile compounds. This process significantly reduces the availability and toxicity of metals in soil and water. Through enzymatic reduction and methylation, bio-volatilization transforms toxic metals into less harmful forms (Tang et al. 2024). Various enzymes are involved in the bio-volatilization of metals such as arsenic (As), mercury (Hg), and antimony (Sb). Notable examples include arsenic methyltransferase for As, mercury reductase for Hg, and antimony methyltransferase for Sb. These metals are converted into non-toxic compounds through this method (Boriová et al. 2014). Bacterial enzymes, particularly methyltransferases, facilitate the conversion of As(V) into mono-, di-, and tri-methylated arsenic species. These methylated forms are volatile, allowing them to be released into the atmosphere. Additionally, research indicates that enzymes such as reductase (MerA) and mercurial lyase, found in archaea and eubacteria, also contribute to bio-volatilization (Freedman et al. 2012). Furthermore, the fungus Scopulariopsis brevicaulis has shown promising results in converting As(V) and Hg(II) into their non-toxic states (Boriová et al. 2014).

**Bioleaching**

Bioleaching is a process carried out by a variety of microorganisms, with acidophiles being the most prominent among them. Acidophiles are chemolithotrophs that oxidize Fe (II) to Fe (III) and/or reduce sulfur to sulfuric acid, thriving in low pH environments, particularly at pH 2.0 or below. The sulfuric acid produced generates ferric ions and protons, which help solubilize metal sulfides and oxides from ores (Srichandan et al. 2014). This process facilitates the extraction of metals by separating them from the less water-soluble solid phase.

In addition to their role in bioleaching, microorganisms can be utilized as reducing agents to extract and recover heavy metals. The effectiveness of this recovery process hinges on the ability of microorganisms to convert solid contaminants in soil into soluble substances that can be removed and recovered. Given that metal resources are non-renewable, recovering metals from industrial wastewater presents a viable option for ensuring a continuous supply of heavy metals. Several researchers have proposed bioremediation as a method for recovering raw materials from effluent (Pande et al. 2022).

For example, using an Annona squamosa-based absorbent combined with 0.1 M HCl, a Cd(II) recovery rate of up to 98.7% was achieved (Isaac and Sivakumar 2013). Additionally, a recovery rate of 82% for Cd(II) was obtained with *Pseudomonas aeruginosa* biomass and 0.1 M HCl. In another study, 100% Cu(II) recovery was reported when using volcanic rock matrix-immobilized P. putida cells displaying cyanobacterial metallothioneins at pH 2.35 (Ni et al. 2012).

#### Phytoremediation

Phytoremediation is a plant-based technique used to remove, degrade, or stabilize contaminants in soil and water. This process involves plants absorbing pollutants into their tissues, precipitating them onto their roots, facilitating their breakdown by associated microorganisms, or stabilizing them into a harmless state through mechanisms such as Phyto immobilization and Phyto stabilization (Raklami et al. 2022). As a remediation strategy, it is operationally simple, aesthetically appealing, cost-effective, and widely accepted. Two significant methods for phytoremediation are phytoextraction and Phyto stabilization. During the process of phytoextraction, plants absorb heavy metals from the soil and accumulate them in their shoots and leaves. Phyto stabilization effectively immobilizes heavy metals in the soil through mechanisms such as root absorption, adsorption onto root surfaces, exudate-induced complexation or precipitation, rhizospheric reduction, and enhancement of soil stability. Plants used for Phyto stabilization exhibit tolerance to heavy metals, possess substantial root biomass and effectively minimize the transport of metals from their roots to the aboveground parts. Sibth, Red Fescue, wiregrass, thatching grass, Syrian bean-caper, and hippo grass demonstrate efficacy in addressing soils contaminated with Pb, Zn, Cr, and Cu.

**Fig. 11:**
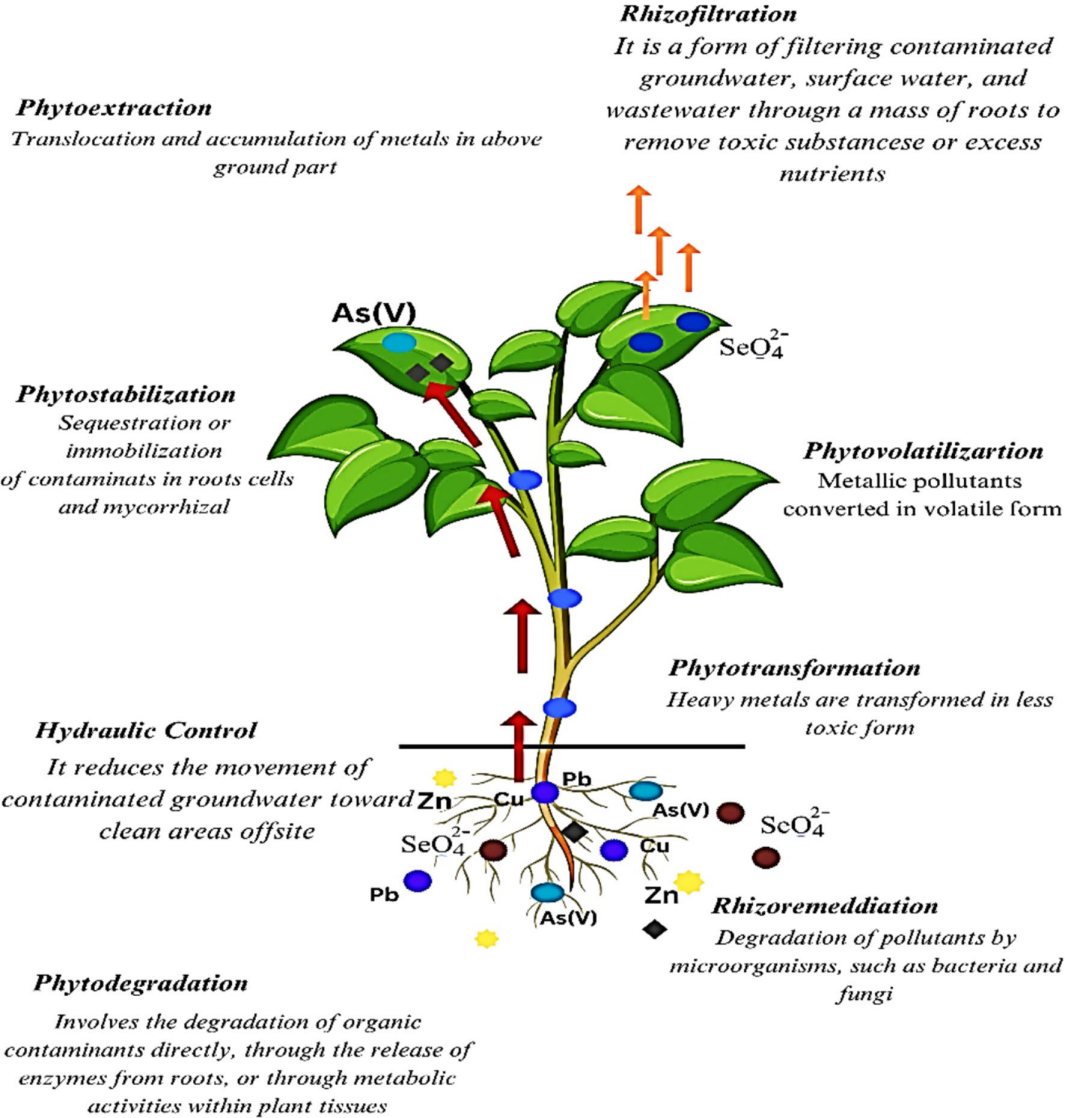
Phytoremediation of heavy metals in plants (Ojuederie and Babalola 2017)

The success of Phyto management depends on selecting suitable plant species that are fast-growing, deeply rooted, easy to propagate, and capable of producing high biomass. Additionally, these plants should be capable of performing various phytoremediation processes, such as phytoextraction, phytodegradation, rhizofiltration, phytostabilization, phytovolatilization, phytotransformation, rhizoremediation, and hydraulic control (Fig. 11) (Rigoletto et al. 2020). These processes allow plants to address soil contamination by either removing, neutralizing, or stabilizing pollutants. Moreover, the selected plants must help mitigate risks associated with the contaminated soil, such as reducing leaching or stabilizing the soil structure. Importantly, the cultivation of these plants should be economically viable and feasible under specific site and land-use conditions (Emamverdian et al. 2015).

##### Trees

Different tree species have the potential to produce biomass in contaminated soil. Willow (Salix spp.) and poplar (Populus spp.) are employed in bioenergy production because of their rapid growth and capacity for coppicing, particularly through short-rotation coppicing methods. Species such as eucalyptus, beech, maple, and birch, along with spruce, pine, fir, larch, and hemlock, serve as sources for pulp, lumber, and fuel. Nonetheless, it is crucial that these trees do not accumulate high levels of trace elements (TE) in the wood, as their release during processing (bioenergy generation, pulp, or combustion) or use (furniture or paper) could pose significant risks. The presence of TE in leaves has the potential to extend its influence into surrounding ecosystems.

Pb is an exception, as certain species such as willow and poplar are known to accumulate high concentrations of Cd and Zn (Yin et al. 2024). Birch, a pioneer tree, is known for its fast growth and low nutrient requirements, making it a suitable candidate for phytomanagement on contaminated soils with low nutrient contents. However, birch tends to accumulate more lead than other tree species, such as willow, poplar, oak (Quercus spp.), beech, and maple (Deighton et al. 2023).

Further research by Saboor et al. (2021) found that the accumulation of Cd and Zn in leaves followed the order: aspen (*Populus tremula*) > silver birch (*Betula pendula*) >  > Scots pine (*Pinus sylvestris*) ≈ oak (*Quercus robur* and *Quercus petraea*). For stems, the accumulation order was: aspen ≈ silver birch > Scots pine > oak. Scots pine is considered a good bioindicator for industrial pollution due to its sensitivity (Usoltsev et al. 2019). Eucalyptus species, such as Eucalyptus camaldulensis, are less effective at accumulating lead (Khamare et al. 2023), though some species like *Eucalyptus polybractea* and *Eucalyptus cladocalyx* have shown the ability to accumulate TE such as Cd and Zn to high levels. Oaks, while tolerant of TE, exhibit low uptake of these elements, which limits their utility for phytomanagement, despite the value of their wood (Budzyńska et al. 2024). Maple (Acer spp.) demonstrates a low tendency to accumulate Zn and Cd, but its accumulation of Pb and chromium (Cr) is comparable to that of willow, poplar, or birch (Emamverdian et al. 2015).

##### Agricultural crop plants

Bioethanol production utilizes crops such as wheat (*Triticum* spp.), maize (*Zea mays* L.), sweet and grain sorghum (*Sorghum bicolor* (L.) Moench), and sugar beet (*Beta vulgaris* L.), due to their capacity to collect substantial quantities of starch or sugars in their storage organs, suitable for fermentation. Annual plants with elevated seed oil content are favored for biodiesel production, including sunflower (*Helianthus annuus* L.), rapeseed (*Brassica napus* L. var. oleifera D.C.), soybean (*Glycine max* L.), and tobacco (*Nicotiana tabacum*). In addition to starch, sugar, and oil-rich plant components, stover and straw may also be used for bioenergy generation.

Soybean (Glycine max), wheat (*Triticum aestivum* L.), and corn (*Zea mays*) seeds accumulate significantly higher amounts of zinc (Zn) than their stems, with soybean accumulating up to six times more Zn (Evangelou et al. 2015). However, no significant differences are observed for cadmium (Cd) and lead (Pb) concentrations between seeds and stems. The production of methane or ethanol through anaerobic digestion requires low concentrations of trace elements (TE), as these elements can negatively impact the enzymes responsible for biomass breakdown and may present challenges regarding the fate of the digestate when applied to soils.

Osman et al. (2024) reported a decrease in As accumulation in sorghum, while sugarcane was found to accumulate approximately 50% less TE in its stalks compared to its leaves, with cadmium (Cd) being an exception, where higher concentrations are found in the leaves (Evangelou et al. 2015). Additionally, Saleem et al. (2024) highlighted that sugarcane (*Saccharum officinarum*) has a high capacity for tolerating and accumulating Cd. Tobacco (Nicotiana tabacum) has been reported to accumulate Cd to relatively high levels compared to other species, with Cd concentrations in field-grown tobacco leaves ranging from 1,000 mg/kg without significant biomass reduction (Liu et al. 2019a; Lu and Lu 2019).

Rapeseed (*Brassica napus*) has been found to accumulate more Pb than wheat, corn, and sorghum (Bosch-Serra et al. 2020). Considering the potential risks associated with biomass produced on contaminated soils, it can be concluded from the available literature that tobacco and sugarcane would be unsuitable for Cd-contaminated soils, soybean for Zn-contaminated soils, and wheat for As-contaminated soils. Furthermore, rapeseed is generally unsuitable for TE-contaminated sites due to its classification in the Brassicaceae family, which includes many hyperaccumulator species (Evangelou et al. 2015).

##### Herbaceous perennial crops

Perennial grasses have long served as a source of fodder and feed for draft animals, playing a significant role in enhancing agricultural energy production. In 1920, the United States supported 27 million animals across 35–40 million hectares of grasslands for both agricultural and urban traction (Curry-Lindahl 2019). Given their bioenergy potential, these perennial grasses could experience a resurgence in the twenty-first century. Switchgrass, miscanthus, reed canary grass, vetiver grass, elephant grass, and gigantic reed represent viable options.

In contrast to trees and crops, there has been limited investigation into the accumulation of trace elements in perennial grasses. Consequently, the optimal perennial grasses for the phytomanagement of TE-contaminated areas have yet to be identified. Evangelou et al. (2015) suggested the use of hybrid Pennisetum, giant reed (*Arundo donax*), silver reed (*Thamnochortus cinereus*), and switchgrass (*Panicum virgatum*) for the phytoextraction of As, Hg, Cu, Cr, Pb, and Cd contaminated soils. The ability of Pennisetum to accumulate Cd was confirmed by Morita et al. (2024), who demonstrated that it contained higher levels of Cd compared to vetiver grass. Giant reed (*Arundo donax*) has the potential to accumulate significant quantities of Cd and Ni trace elements (Liu et al. 2019b).

## Challenges of using microorganisms as bioremediation

### Metal toxicity to microorganisms

High concentrations of heavy metals, such as cadmium (Cd), lead (Pb), mercury (Hg), and arsenic (As), can inhibit microbial growth and metabolism, thereby reducing the efficiency of bioremediation. Microorganisms must either possess or be engineered to have high metal resistance to survive and function effectively in contaminated environments (Fashola et al. 2016).

### Environmental variability

Factors such as pH, temperature, salinity, oxygen availability, and the presence of co-contaminants significantly affect microbial activity and metal bioavailability. These variables make it challenging to predict or control microbial performance in the field (Gupta et al. 2021).

### Limited field applications

Successes observed in laboratory settings often do not translate to field conditions due to an insufficient understanding of the interactions between soil, microbes, and metals, as well as microbial survival under complex environmental stressors (Qattan 2025). Additionally, challenges concerning scale-up, regulatory frameworks, and site heterogeneity remain unresolved.

### Horizontal gene transfer and biosafety risks

Some metal-resistant microbes carry resistance genes on mobile genetic elements, raising the risk of horizontal gene transfer to pathogenic microbes. This poses biosafety concerns, particularly when using genetically modified organisms (GMOs) (Ashraf et al. 2022).

### Competition with native microflora

Introduced microbial strains often compete with indigenous microbiota, which can limit their survival, colonization, and bioremediation capabilities. Native microbes may also disrupt engineered microbial consortia (Chatterjee et al. 2023).

## Nano remediation of heavy metals

Nano remediation of heavy metals is an emerging approach in environmental management that utilizes nanotechnology to tackle heavy metal pollution. Nanotechnology, derived from the Greek word "Nanos," meaning "small," involves manipulating materials at the atomic or molecular scale, typically ranging from 1 to 100 nm. This technology has gained significant attention in agriculture for enhancing plant stress tolerance and improving remediation strategies. Various nanoparticles (NPs), such as ZnO-NPs, Zn-NPs, Ag-NPs, FeS₂-NPs, Fe-NPs, CeO₂-NPs, SiO₂-NPs, Au-NPs, TiO₂-NPs, CuO-NPs, and carbon-based NPs like carbon nanotubes and fullerols, have been tested in controlled and natural field conditions to help plants survive heavy metal stress and facilitate remediation (Ullah et al. 2023). These nanoparticles exhibit unique properties such as high surface area, reactivity, and mobility, making them effective in addressing heavy metal contamination.

Nano remediation works through several mechanisms, including the adsorption of heavy metals from soil or water by nanoparticles, thus preventing their absorption by plants or immobilizing them in a non-toxic form. Some nanoparticles, such as iron nanoparticles (Fe-NPs), can facilitate the uptake and sequestration of heavy metals in plant tissues in a less toxic or bioavailable form. Furthermore, nanomaterials can enhance phytoremediation processes, where plants absorb and accumulate contaminants. By promoting plant growth, improving nutrient uptake, and stimulating antioxidant systems, nanoparticles help mitigate the toxic effects of heavy metals on plants (Ullah et al. 2023). The high surface area to volume ratio and the ability to be functionalized for specific applications make nanoparticles ideal for improving heavy metal bioavailability, plant uptake, and detoxification processes. This approach, therefore, offers a promising, environmentally resilient strategy for the management and remediation of heavy metal pollution in agricultural environments (Table 3).

**Table 3:** Effect of different nanoparticles (NPs) used in different plant species under heavy metal toxicity

NP	NP concentration	Plant species	Effects	References
Arsenic (As)				
Zinc oxide nanoparticles	10, 20, 50, 100 mg/L	*Oryza sativa*	Enhanced the growth and photosynthesis of rice seedlings. Phytochelatins (PCs) content up-regulated in the roots induced more As (III)-PC to be complexed and reduced As (III) mobility for transport to shoots	Yan et al. (2021)
	10 ppm	*Luffa acutangula*	Enhanced photosynthetic pigments, proline, relative water content, total sugars, proteins and indole acetic acid and reduced lipid peroxidation and electrolyte leakage	Tanveer et al. (2022)
	30 mg/L	*Oryza sativa*	Reduced As accumulation, scavenge reactive oxygen species (ROS) like hydrogen peroxide (H_2_O_2_) and superoxide anion (O_2_^.−^), while significantly promoted the gas exchange parameters, chlorophyll content (SPAD value), fluorescence efficiency (Fv/m) and antioxidant enzyme	Jalil et al. (2023)
Molybdenum nanoparticles	100 mg/L	*Triticum aestivum*	Increased biomass and height, improved ion balance, and decreased As absorption	Ahmed et al. (2022)
Magnesium oxide nanoparticles	50–200 mg/L	*Glycine max Oryza sativa*	Improved biomass, photosynthesis, nutrient uptake, and activity of catalase and superoxide dismutase, and decreased As, lipid peroxidation, and hydrogen peroxide accumulation	Faizan et al. (2022) Ahmed et al. (2021)
Calcium oxide nanoparticles	25 mg/L	*Hordeum vulgare*	Increased growth and biomass, decreased As accumulation and reactive oxygen species, upregulated the activity of catalase, peroxides and superoxide dismutase	Nazir et al. (2022)
Iron oxide nanoparticles	500 mg/L	*Brassica juncea*	Increased germination and decreased the activity of catalase, superoxide dismutase, and ascorbate peroxidase	Praveen et al. (2018)
Titanium dioxide nanoparticles	25 and 50 mg/L)	*Oryza sativa*	Upregulating the activity of antioxidant enzymes and glyoxalase cycle decreased hydrogen peroxide, methylglyoxal, malondialdehyde, and electrolyte leakage, and thus protected the photosynthetic apparatus and increased plant growth	Kiany et al. (2022)
Cadmium (Cd)				
Zinc oxide nanoparticles	25, 50, 100 mg/L	*Triticum aestivum*	Improved yield, leaf chlorophyll contents and also reduced the oxidative stress, Cd content and increased the leaf superoxide dismutase and peroxidase activities	Adrees et al. (2021)
Iron oxide nanoparticles	10 mg/L	*Phaseolus vulgaris*	Enhanced accumulation of K + content, increased antioxidant defense system, and higher spermidine (Spd) and putrescine (Put) levels	Koleva et al. (2022)
Silicon nanoparticles	20 mg/L	*Phaseolus vulgaris*	Improved potassium content, biosynthesis of polyamines (PAs), and decreased malondialdehyde (MDA) content and electrolyte leakage (EL)	Koleva et al. (2022)
Zinc oxide nanoparticles	10 ppm	*Zea mays*	Improved relative water content, photosynthetic pigments, proline, total sugars, and proteins and decreased lipid peroxidation	Shafiq et al. (2022)
Titanium dioxide nanoparticles	10 ppm			
Selenium nanoparticles	20, 40, and 60 mg/L	*Coriandrum sativum*	Increased shoot and root weight, chlorophyll (Chl), relative water content, phenolic and flavonoid contents and decreased lipid peroxidation	Babashpour-Asl et al. (2022)
Silver nanoparticles	40 mg/L	*Daucus carota*	Reduced reactive oxygen species (ROS), lipid peroxidation (MDA), and Cd uptake, and increased growth, chlorophyll, and antioxidant activity	Faiz et al. (2022a, b)
Lead (Pb)				
Silver nanoparticles	10 ~ 50 mg/L	*Vigna radiate*	Enhanced ionic homeostasis, growth, biomass, yield, photosynthetic rate, total chlorophyll, water use efficiency, and antioxidant enzyme activity Reduced ROS concentration, MDA, and Pb uptake	Chen et al. (2022)
Titanium dioxide nanoparticles	5 mg L^−1^	*Lactuca sativa*	Increased growth and gas exchange parameters, and decreased Pb uptake, MDA, and ROS	Mariz-Ponte et al. (2021)
Magnesium oxide nanoparticles	(5 mmol/L	*Daucus carota*	Increased the activity of antioxidant enzymes including superoxide dismutase (SOD) and Catalase (CAT), increased concentration of iron, manganese, copper, and zinc	Faiz et al. (2022a, b)
Chromium (Cr)				
Zinc oxide nanoparticles	50 ~ 100 mg/L	*Oryza sativa*	Increased growth, photosynthetic efficiency, nutrient uptake, activity, and expression of antioxidative enzymes, and reduced Cr uptake, MDA, and ROS content	Prakash et al. (2022)
Copper nanoparticles	25–50 mg/kg	*Triticum aestivum*	Increased biomass, proline, total phenolics, antioxidant enzyme activity, and Cr-immobilization in the soil and reduced MDA, H_2_O_2_, and Cr buildup in the shoot and root	Noman et al. (2020)

### Remediation of heavy metals by functionalized carbon nanoparticles

Scientists are increasingly using specially designed carbon nanoparticles to capture harmful metals from wastewater. These nanoparticles boast unique properties, making them efficient filters for water treatment (Elsehly et al. 2024). Different types of carbon nanomaterials, including carbon nanotubes and graphene, are being explored for this purpose (Kumar 2023). Graphene, a super-thin sheet of carbon atoms, is particularly promising due to its large surface area and the presence of oxygen groups that make it water-soluble (Abdel Wahab et al. 2023). This property is especially valuable for water treatment applications. Graphene oxide (GO), a derivative of graphene, is another star player with its high surface area and diverse functional groups that effectively capture pollutants (Ramanathan et al. 2024; Tong et al. 2024). Overall, these functionalized carbon nanoparticles hold significant potential for cleaning up contaminated water. Besides water cleaning it is also use in gene delivery, biosensors, drug delivery, cancer therapy, phytothermal therapy and regenerative medicines (Filippi et al. 2021).

### Remediation of heavy metals by zeolites nanoparticles

Zeolite materials are like microscopic sponges with a rigid internal structure. Imagine a network of tiny tunnels and cavities built from silicon and aluminum oxide building blocks. These channels are just the right size to trap positively charged atoms (cations) like sodium, magnesium, potassium, and calcium (Barhoum et al. 2022). The zeolite's negative charge attracts these positive ions, holding them within the structure. Studies show that synthetic zeolites are more effective than natural ones at removing metal ions from water (Senila et al. 2024). For instance, research by Tufail et al. (2025) demonstrated that zeolite nanoparticles embedded in special membranes could remove lead and nickel from water solutions. This approach not only improves metal removal but also reduces pressure on the membranes. The study also mentions another method for water purification using forward osmosis (FO) membranes. Jain et al. (2021) created an ultrathin FO membrane by combining cellulose acetate and titanium dioxide nanoparticles. This membrane showed high water permeability and stability, making it efficient for desalination (removing salt from water).

### Remediation of heavy metals by Polymer based nanomaterials

Researchers are excited about using nanofibrous membranes made from natural materials like cellulose and chitosan to clean up water. These membranes are considered eco-friendly because they are biodegradable (break down naturally). Their secret lies in a network of tiny pores and a large surface area, allowing them to grab onto pollutants like a sponge (Jahan et al. 2022; Baig et al. 2022). Scientists can also customize these membranes to target specific pollutants by adding special functional groups (Asghar et al. 2024). Overall, these nanofibrous membranes offer a promising and sustainable approach for water purification.

#### Cellulose based nanoparticles

Scientists are exploring the use of cellulose, a natural biopolymer, to create materials that remove harmful heavy metals from water (Mazibuko et al. 2024). Several studies have shown promising results. For example, Azimi et al. (2024) developed cellulose nanofibers that effectively captured lead and copper ions from water. Salem et al. (2024) also demonstrated success using cellulose nanofibers modified with phosphate groups to remove copper ions. Another approach involved modifying cellulose nanofibers with thiol groups, which showed good adsorption capacity for lead, cadmium, and copper (Aljohani et al. 2024).

#### Dendrimers based nanoparticles

Scientists are developing a promising method for water purification that uses specially designed organic polymers called dendrimers. These polymers have functional groups that can grab onto and hold toxic metals (Mohamed et al. 2024a, b). Researchers have created a type of dendrimer material that looks like a gel and can remove several metals from water, including cadmium, nickel, copper, and cobalt (Jacinto et al. 2024). However, this material seems to be more effective at capturing copper and cobalt compared to cadmium and nickel (Nyabadza et al. 2024). Another study explored nanofibers made from a combination of polyacrylonitrile, diethylenetriamine, ethylene glycol, and ethylenediamine (Wang et al. 2024). These nanofibers showed impressive adsorption capacity for zinc, lead, and cadmium ions, reaching 7.2, 8.8, and 6.1 mmol/g respectively. This high capacity is attributed to the large surface area of the nanofibers, which allows for more interaction between the functional groups and the targeted heavy metals.

### Chitosan based nanoparticles

Chitosan nanoparticles, derived from natural sources like shrimp shells, are attracting attention for their potential in water purification (Lakshmi et al. 2024). These nanoparticles are eco-friendly (biodegradable, non-toxic) and offer several advantages like water solubility and the ability to be modified to target specific pollutants. Chitosan's structure, rich in amine and hydroxyl groups, allows it to act as a chelating agent, grabbing onto heavy metals and dyes (Benettayeb et al. 2024). Scientists are exploring different ways to modify chitosan to enhance its effectiveness. For instance, Bessa et al. (2020) created chitosan-based carbon nanoparticles that showed impressive capacity for removing mercury from water. Other studies have combined chitosan with other materials like zinc oxide (Khalid et al. 2022) to create nanocomposites effective against various heavy metals.

### Magnetic nanoparticles

**Fig. 12:**
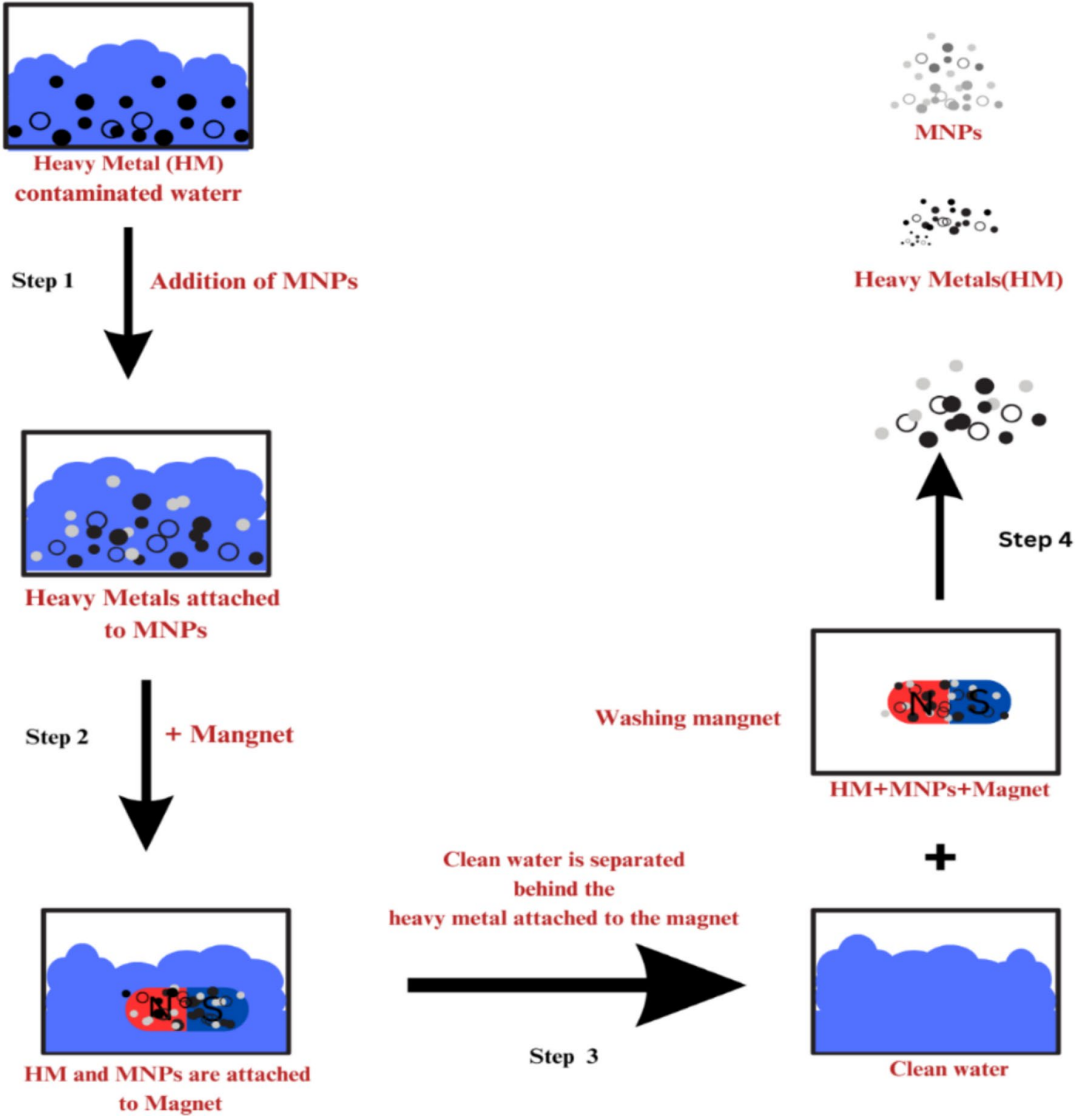
An overview of water purification utilizing magnetic nanoparticles

Magnetic nanoparticles are a powerful tool for cleaning up polluted water, especially when it comes to removing heavy metals (Liosis et al. 2021). These tiny particles have a large surface area that efficiently grabs onto heavy metals. Another major advantage is that they are magnetic, which allows for easy separation from the treated water (Fig. 12). Iron oxide nanoparticles are a popular choice for water purification because they can be easily reused, separated from treated water, and hold a significant amount of heavy metals (Yadav et al. 2023). Studies have shown success using these nanoparticles to remove lead, copper, manganese, and zinc ions from water (Geioushy et al 2024; Sharma et al. 2024a, b). The effectiveness of this method can be influenced by various factors, such as the interaction between the nanoparticles and the specific metal being targeted.

## Limitations of key remediation approaches

### Phytoremediation

#### Challenges

Slow process requiring multiple growing seasons.

Limited to surface and root-zone contamination (shallow depths).

Dependent on plant species and local climate.

Risk of contaminant transfer into the food chain.

Biomass disposal of contaminated plants remains problematic.

### Field-Scale Barrier

Poor adaptability to highly polluted or nutrient-poor soils.

Limited efficacy under extreme weather or salinity conditions.

### Microbial Bioremediation

#### Challenges

Metal toxicity can suppress microbial activity.

Environmental parameters (pH, temperature, moisture) greatly influence efficacy.

Competition with native microbiota and low survivability in field settings.

Regulatory hurdles in using genetically modified microbes.

### Nanoremediation

#### Challenges

High cost of nanoparticle synthesis.

Potential ecotoxicity of nanoparticles.

Limited data on long-term stability and fate of nanomaterials in soil.

### Field barrier

Limited field validation and potential regulatory restrictions due to environmental safety concerns.

### Key insights and recommendations

Integration Over Isolation: Relying on a single remediation method is often insufficient. Integrating phytoremediation with microbial consortia or biochar amendments can enhance efficiency and resilience.

Focus on Field-Scale Validation: Many approaches remain at the proof-of-concept stage. More long-term, site-specific pilot studies are needed to evaluate sustainability and effectiveness under variable environmental conditions.

Economic Considerations: While nanotechnology offers high removal efficiency, cost and potential ecotoxicity may limit its practicality. Low-cost, natural amendments (e.g., compost, biochar) remain more accessible in developing regions.

Monitoring and Risk Assessment: Implementation should be accompanied by thorough risk assessments, particularly when using nanoparticles or genetically modified organisms, to ensure safety and regulatory compliance.

## Bioremediation and its role in sustainable agriculture and soil management policies

Bioremediation of heavy metals plays a crucial role in promoting sustainable agriculture by restoring soil health, enhancing crop productivity, and reducing environmental degradation. By utilizing the natural or engineered capabilities of microorganisms and plants to detoxify metal-contaminated soils, bioremediation provides a regenerative approach that aligns with sustainable land-use and agroecological practices.

### Restoring soil fertility and function

Heavy metal pollution significantly degrades soil microbial diversity, structure, and nutrient cycling. Bioremediation—particularly through the use of microbial consortia and biochar-assisted strategies—can restore microbial activity, enzymatic functions, and the essential balance of nutrients, thereby rehabilitating soils for productive use (Islam et al. 2022). This not only reduces reliance on chemical inputs but also enhances long-term soil resilience.

### Reducing health risks in food chains

Heavy metals can accumulate in edible plant parts, posing serious health risks to humans and livestock. By decreasing metal bioavailability through microbial immobilization or phytostabilization, bioremediation helps prevent the transfer of contaminants into the food chain. This is essential for meeting food safety standards under policies such as the EU Soil Strategy and Codex Alimentarius (Bolan et al. 2023).

### Supporting low-input and circular farming systems

Bioremediation technologies—especially those that employ compost, microbial inoculants, and phytoremediation cover crops—can be integrated into low-input or organic farming systems. This promotes resource recycling and aligns with the principles of a circular economy in agriculture, particularly in developing countries facing fertilizer shortages (Khan et al. 2022a, b).

### Policy implications and guidelines

Incorporating bioremediation into soil management policies can support national and international initiatives such as the UN Sustainable Development Goals (SDG 2 & SDG 15) by:

- Encouraging the adoption of bioremediation for reclaiming marginal lands.
- Providing incentives for the use of bio-based amendments in soil restoration.
- Regulating the safe use of biosolids and GMOs for site-specific remediation (Rodríguez et al. 2024).
- Governments and agencies can facilitate this integration by:
- Establishing field-scale validation programs.
- Developing bioremediation risk assessment frameworks.
- Promoting farmer training and participatory soil remediation programs.

## Conclusions

**Fig. 13:**
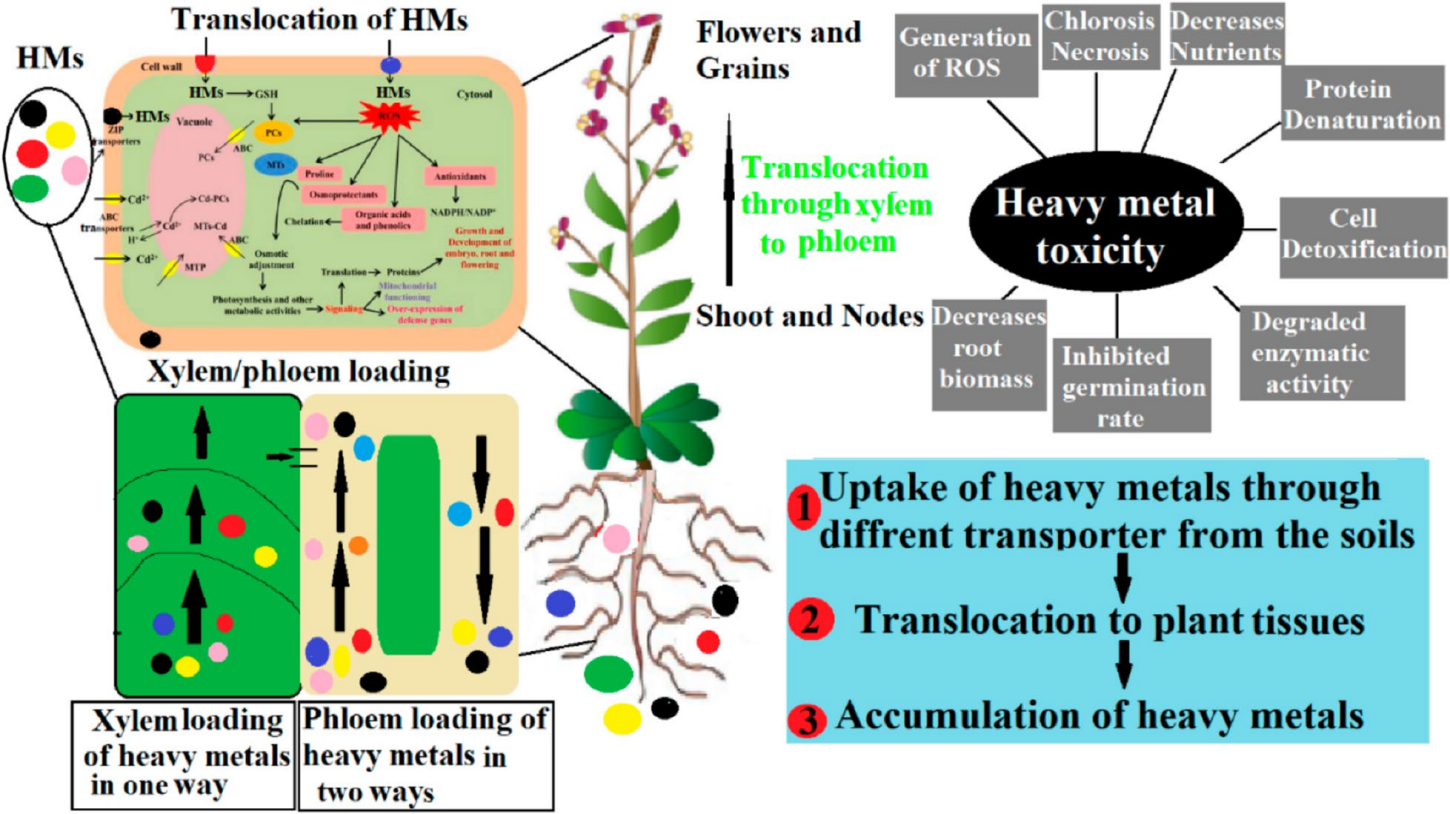
Summary of heavy effects and translocations

Climate change, low agricultural crop productivity, abiotic stressors, pollution of the environment and soil, and startled plant growth are the main issues facing the agriculture sector. Climate change significantly impacts heavy metal toxicity and remediation in plants. Rising temperatures, altered precipitation, and increased atmospheric CO₂ affect the bioavailability of heavy metals, plant uptake, and remediation efforts. Soil temperature and moisture fluctuations can enhance the mobility of metals like cadmium, arsenic, and lead, increasing their bioavailability. Drought stress, common under climate change, diminishes a plant's detoxification capacity by impairing antioxidant activity and root metabolism. Elevated CO₂ levels may improve plant biomass and root exudation, potentially enhancing phytoremediation, although results vary by species and metal type. Changes in microbial activity can also affect the rhizosphere, influencing bioremediation processes. Furthermore, climate stressors may disrupt gene expression related to metal transport and antioxidant systems, reducing plant resilience. Integrating climate resilience into remediation strategies is essential for sustaining agricultural productivity and ecosystem health in metal-contaminated environments. To attain sustainable crop production and food security, scientists are utilizing technology and measures to reduce these obstacles. Among the many novels, cutting-edge procedures, one that is environmentally resilient is heavy metals remediation. The mechanism of action of heavy metals is to disrupt cellular functions, which ultimately results in cell death. The mechanism of action of heavy metals begins when roots take up these toxicants, grow larger, upset the nutrient-water balance, inactivate vacuoles, and experience a drop-in mitotic activity, all of which inhibit root growth. They disturb cellular processes, metabolic pathways, cellular ion concentration imbalances, and alterations in gene expression that result in aberrant proteins by penetrating the cells through transport channels and interfering with enzymatic activity. Hyperaccumulators take up heavy metals from soils with changed pH levels, organic matter, and water contents by releasing the metals, mobilizing them, and allowing roots to absorb them. These heavy metals are then carried across the xylem by a variety of proteins, including those that carry ATPases, Nramp, ZIP, and MATE (Fig. 13). They disturb cellular processes, metabolic pathways, cellular ion concentration imbalances, and alterations in gene expression that result in aberrant proteins by penetrating the cells through transport channels and interfering with enzymatic activity. Hyperaccumulators take up heavy metals from soils with changed pH levels, organic matter, and water contents by releasing the metals, mobilizing them, and allowing roots to absorb them. These heavy metals are then carried across the xylem by a variety of proteins, including those that carry ATPases, Nramp, ZIP, and MATE. Microbial bioremediation, phytoremediation, mycoremediation, bioaugmentation, and biosorption are examples of bioremediation. Microorganisms carry out microbial bioremediation to break down organic waste. In phytoremediation, which is accomplished by phytoextraction and phytostabilization, contaminants are absorbed by plants through immobilization in the soil and roots, whereupon they are broken down by microbes. Utilizing nanotechnology, carbon, zeolites, magnetic nanoparticles, polymers, cellulose, dendrimers, and chitosan-based nanoparticles, nano remediation is the most recent technique for eliminating heavy metals from the environment. The magnetic nanoparticle technique, which employs the magnetic field of nanoparticles from heavy metal-contaminated water to attract heavy metals and clean the water, is quite unique among nano remediation techniques.

## Data Availability

Not applicable.
